# Amphiphilic Diblock Copolymers of Poly(N-vinyl pyrrolidone) and Poly(vinyl esters) Bearing N-Alkyl Side Chains for the Encapsulation of Curcumin and Indomethacin

**DOI:** 10.3390/polym17212852

**Published:** 2025-10-26

**Authors:** Nikolaos V. Plachouras, Aikaterini-Maria Gkolemi, Alexandros Argyropoulos, Athanasios Bouzoukas, Theodosia-Panagiota Papazoglou, Nikoletta Roka, Marinos Pitsikalis

**Affiliations:** Industrial Chemistry Laboratory, Department of Chemistry, National and Kapodistrian University of Athens, Panepistimiopolis Zografou, 15771 Athens, Greece; plachouras.v.nikolaos@gmail.com (N.V.P.); katerinagolemi228@gmail.com (A.-M.G.); argiropoulosal@gmail.com (A.A.); thanasisbuzukas@gmail.com (A.B.); pnpapazoglou@gmail.com (T.-P.P.); nikolettaroka@yahoo.gr (N.R.)

**Keywords:** amphiphilic block copolymers, poly(N-vinyl pyrrolidone), poly(n-alkyl vinyl esters), self-assembly, polymeric micelles, drug encapsulation, curcumin, indomethacin, light scattering

## Abstract

Τhe self-assembly behavior of a series of amphiphilic diblock copolymers, each consisting of a hydrophilic poly(N-vinyl pyrrolidone) (PNVP) block and a hydrophobic block derived from n-alkyl vinyl esters, namely poly(vinyl butyrate) (PVBu), poly(vinyl decanoate) (PVDc), and poly(vinyl stearate) (PVSt), in aqueous solutions was investigated. Dynamic and static light scattering (DLS and SLS) techniques were employed to monitor the micellization behavior. In addition, the self-assembled structures were observed with Transmission Electron Microscopy (TEM). The effect of the nature of the hydrophobic block, the copolymer composition and the copolymer molecular weight on the self-assembly properties was thoroughly examined. The encapsulation of curcumin and indomethacin within the dry cores of the micellar structures was conducted in aqueous solutions for all block copolymers at various curcumin/indomethacin-to-polymer mass ratios. UV-Vis spectroscopy was used to evaluate the drug-loading capacity and efficiency (%DLC and %DLE). In several cases, the encapsulation of both hydrophobic drugs was found to be nearly quantitative. Combined with the observed stability of the micellar structures, these findings suggest that the block copolymers demonstrate significant potential as carriers for drug delivery applications.

## 1. Introduction

A major obstacle in the efficient development of pharmaceuticals is still their limited solubility in aqueous media. Despite efforts to address this issue, over 40% of licensed medications and up to 90% of investigational substances exhibit low solubility in water, according to estimates [1]. The proliferation of barely water-soluble drug candidates, which is attributed to the strategic application of combinatorial chemistry and the routine implementation of high-throughput screening in non-aqueous systems [2,3], is typically accompanied by a range of associated problems. Their poor water solubility lowers their pharmacological potential by restricting dissolution and absorption, promoting aggregation or premature precipitation in aqueous environments that may result in recrystallization and variable pharmacokinetics, and facilitating rapid systemic clearance through tissue distribution, plasma protein binding, or accelerated elimination. Furthermore, their clinical activity is further limited by intrinsic chemical and oxidative instability before they reach the target site [4,5,6,7].

An effective strategy to overcome the aforementioned limitations is the employment of biocompatible amphiphilic block copolymers, capitalizing on their spontaneous self-assembling into micelles in aqueous solutions. The hydrophobic core of the micelles can encapsulate therapeutic molecules that would otherwise be insoluble in water, enhancing their apparent solubility and preventing the degradation of the active ingredient [8,9]. Moreover, the hydrophilic shell of the micelles confers colloidal stability, extending their circulation half-life and biodistribution. The tunable architecture of block copolymers enables high drug loading and controlled release profiles. This can be achieved by adding functional groups to target specific tissues or tumors or by including pH- or redox-responsive functionalities [10,11,12]. Finally, the broad spectrum of available loading methods, including thin-film hydration, solvent evaporation, dialysis and co-solvent methods, facilitates optimization of micellar formulation for a wide variety of hydrophobic drugs [13].

**Scheme 1:**
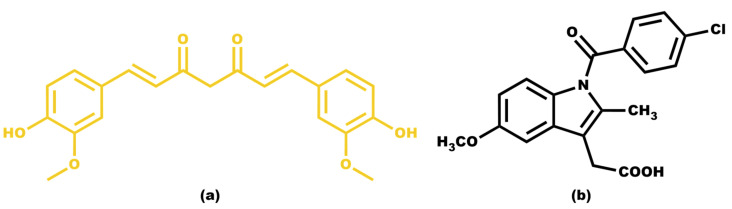
(**a**) Chemical structure of the hydrophobic drug compound curcumin; (**b**) chemical structure of the hydrophobic drug compound indomethacin.

Curcumin is a highly hydrophobic molecule with extremely low water solubility, which limits its bioavailability. It is considered a natural ingredient that has sparked scientific interest due to its numerous advantages for human health, as it is believed to have anti-inflammatory, antioxidant, antibacterial, antiviral, antifungal, antidiabetic, and anticancer properties [14]. Another practically water-insoluble molecule with particularly interesting pharmacological properties is indomethacin. It belongs to the class of nonsteroidal anti-inflammatory drugs (NSAIDs) and has been clinically employed for the treatment of acute pain, rheumatoid arthritis, ankylosing spondylitis, osteoarthritis, bursitis, gouty arthritis, patent ductus arteriosus, and certain types of migraine [15]. Both curcumin and indomethacin can easily be traced by UV-Vis spectroscopy since they exhibit a maximum absorption at 429 nm [16] and 318 nm [17], respectively. The chemical structures of both curcumin and indomethacin are shown in Scheme 1.

**Scheme 2:**
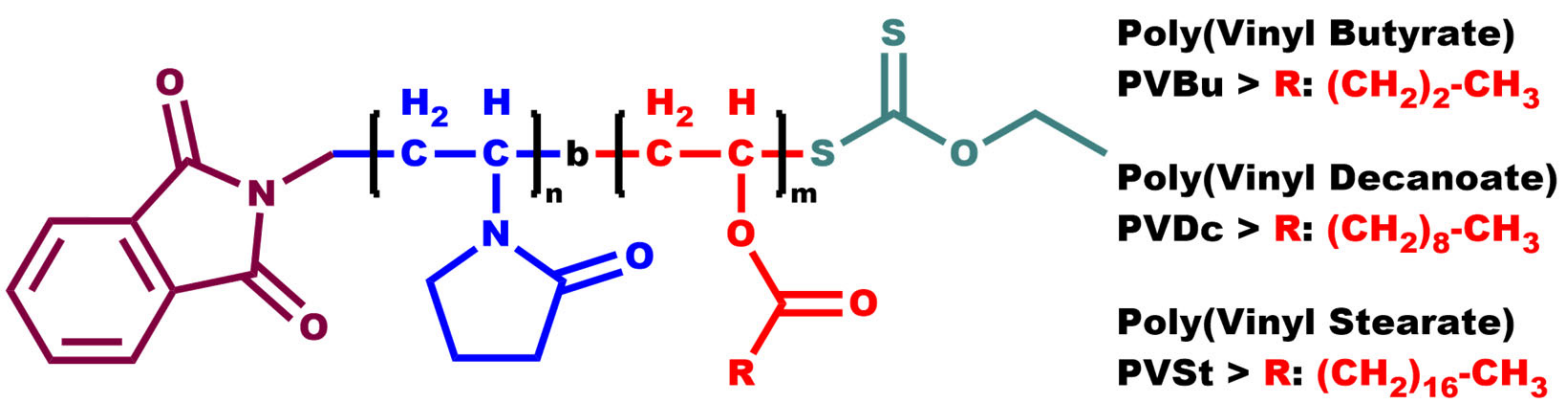
Chemical structures of the amphiphilic diblock copolymers consisting of a hydrophilic PNVP block and a hydrophobic block derived from PVBu, PVDc and PVSt.

The micellization behavior of a series of amphiphilic diblock copolymers, each consisting of a hydrophilic poly(N-vinyl pyrrolidone) (PNVP) block and a hydrophobic block derived from n-alkyl vinyl esters, namely poly(vinyl butyrate) (PVBu), poly(vinyl decanoate), (PVDc) and poly(vinyl stearate) (PVSt), in aqueous solutions was investigated. The chemical structures of each different family of amphiphilic diblock copolymers are given in Scheme 2. Poly(N-vinyl pyrrolidone) possesses distinctive properties, including biocompatibility and non-toxicity, which have led to its extensive industrial utilization in the pharmaceutical and biomedical sectors, as well as others [18]. In fact, recent studies suggest PNVP as the optimal choice for replacing poly(ethylene oxide) (PEO), which is currently the most widely used hydrophilic material employed in the aforementioned sectors [19,20]. Poly(vinyl esters) (PVEs) represent another significant class of polymeric materials, which exhibit either hydrophilic or hydrophobic characteristics, depending on the nature of the side group. These materials are of pharmacological significance due to their capability to serve as precursors for the production of poly(vinyl alcohol) (PVA) through the hydrolysis of their ester group [21,22].

The micellization behavior of amphiphilic block copolymers is governed by a delicate balance of molecular and environmental parameters. The nature of the hydrophobic block is a crucial molecular factor. Flexible and amorphous hydrophobic blocks frequently result in equilibrium micelles, while more rigid and crystallizable blocks favor highly ordered aggregates due to restricted chain mobility [23,24,25]. The length of the alkyl side chain within the hydrophobic block has been demonstrated to further modulate core packing and stability. It has been shown that longer alkyl chains markedly reduce the critical micelle concentration (CMC) and promote the formation of larger, more stable aggregates, as the increased hydrophobic interactions drive self-assembly [26,27,28,29]. The composition of the diblock copolymer also determines the resulting morphology. Specifically, the hydrophilic-to-hydrophobic ratio affects the formed micellar structure. Higher percentage of the hydrophilic block promote spherical micelles, intermediate ratios yield cylindrical or worm-like micelles, and higher percentages of the hydrophobic block lead to the formation of vesicular or lamellar structures [30,31,32]. Finally, the molecular weight of the amphiphilic block copolymers affects both thermodynamics and kinetics of micellization behavior. Higher molecular weights, especially of the hydrophobic block, lower the CMC and enable the formation of well-defined, stable micelles with improved thermodynamic stability [33,34,35]. The targets of the present work are summarized as follows. (a) A study of the micellization behavior of the PNV-b-PVEs block copolymers in aqueous solutions. Water is a selective solvent for the PNVP blocks [36,37,38]. The self-assembly behavior of the same series of diblock copolymers in tetrahydrofuran (THF) has already been presented [39]. Parameters, such as the nature of the PVEs block, the composition and the molecular weight of the block copolymers, are expected to play a crucial role in defining the self-assembly behavior of the amphiphilic block copolymers in water. (b) Application of the supramolecular structures as vehicles for the encapsulation of hydrophobic compounds. Two hydrophobic drugs, curcumin and indomethacin, will be tested for encapsulation into the core of the micellar structures that are formed. The drug loading capacity and efficiency (%DLC and %DLE) values [40] will be calculated. The influence of the nature of the hydrophobic block along with the molecular characteristics of the block copolymers will be examined.

## 2. Materials and Methods

### 2.1. Synthesis of Diblock Copolymers PNVP-b-PVBu, PNVP-b-PVDc and PNVP-b-PVSt

The synthesis of the PNVP-based amphiphilic diblock copolymers has already been published [41]. In summary, the synthesis of the copolymers was accomplished through the implementation of the reversible addition-fragmentation chain transfer (RAFT) radical polymerization technique, in conjunction with the sequential addition of monomers. In each instance, the PNVP block was synthesized first and, after purification and characterization, was used as a macro chain transfer agent (macro-CTA) for the polymerization of each PVE block. For each of the three distinct copolymer classes, designated PNVP-b-PVBu, PNVP-b-PVDc, and PNVP-b-PVSt, a total of four or five different copolymers with varying molecular weights and molar ratios of each block were synthesized in order to investigate the effect of these parameters on the micellization behavior of the products in aqueous solutions. The molecular characteristics of the PNVP-b-PVEs diblock copolymers are provided in Table 1, whereas more details regarding the synthesis and characterization are provided in the Supplementary Material File S1 of the Supporting Information Section (SIS).

### 2.2. Dynamic and Static Light Scattering (DLS and SLS) Techniques

#### 2.2.1. Preparation of Stock Solutions for the DLS and SLS Measurements

The solvent exchange method was selected for the preparation of stock solutions for each of the distinct samples in each of the three different types of diblock copolymers. Specifically, 20 mg of each sample was initially dissolved in 5 mL of dry THF in a vial. The THF had been dried overnight over sodium and distilled prior to use. The samples were left in THF overnight to ensure complete dissolution of the diblock copolymers. Thereafter, 20 mL of extra pure water was added to each vial. Subsequently, the solutions were heated at 65 °C to guarantee full evaporation of the THF, thereby enabling the self-assembly of the amphiphilic diblock copolymers in water. The initial concentrated solution of each sample, with a concentration of about 10^−3^ g/mL, was further diluted to different lower concentrations. For the SLS measurements, the stock solutions of each sample were prepared via the exact same procedure. The examined concentrations for all samples ranged from 0.8 × 10^−4^ up to 1.5 × 10^−3^ g/mL.

#### 2.2.2. Instrumentation and Methods for the DLS and SLS Measurements

Both DLS and SLS measurements were performed on a NanoBrook Omni system from Brookhaven Instruments operating at λ = 640 nm. A JDS Uniphase 22 mW He–Ne laser was used as the light source.

For the DLS experiments, correlation functions were collected at 25 °C at a 90° scattering angle and analyzed by the cumulant method and the CONTIN software [42]. Measurements were carried out ten times for each concentration of each sample and were averaged. Before each measurement, all solutions were filtered using a 25 mm PVDF hydrophilic welded syringe filter with 0.45 μm pore size and directly introduced into the scattering cell. SLS experiments were conducted at the same temperature and scattering angle.

The intensity time autocorrelation function, $g_{2}(q,t)$, was determined following standard procedures, where q is the scattering vector, t the time, and $t_{0}$ the lag time. The scattering vector was defined as $q=\frac{4\pi n_{0}}{\lambda }\sin\left(\frac{\theta }{2}\right)$, with θ being the observation angle, $n_{0}$ the refractive index of the solvent, and λ the wavelength of the incident light.

As mentioned, the autocorrelation functions were analyzed using the cumulant method. The normalized electric field autocorrelation function can be expressed as $g_{1}\left(t\right)=\int_{0}^{\infty }{G\left(\Gamma \right)e}^{-\Gamma t}d\Gamma$, where $G\left(\Gamma \right)$ is the distribution of decay rates and Γ the average decay rate. In the cumulant expansion, this is written as $g_{1}\left(t\right)=exp(-\Gamma t+\frac{\mu_{2}}{2!}t^{2}-\;\frac{\mu_{3}}{3!}t^{3}+\cdots )$, where Γ is the average decay rate of the correlation function and $\mu_{2}$ is the second cumulant describing the variance of the distribution. The polydispersity index (PDI) was calculated as $PDI=\frac{\mu_{2}}{\Gamma^{2}}$, providing a quantitative measure of the distribution width of the relaxation times. From the mean decay rate, the apparent translational diffusion coefficients were obtained. Extrapolation to zero concentration yielded $D_{0,app}$, while the apparent hydrodynamic radii, $R_{h}^{0}$, were obtained from the Stokes–Einstein relation: $R_{h}^{0}=\frac{kT}{6\pi \eta_{S}D_{0,app}}$, where k is the Boltzmann constant, T the absolute temperature, and $\eta_{s}$ the solvent viscosity. The concentration dependence of the diffusion coefficient was described by $D=D_{0,app}(1+k_{D}c)$, where $k_{D}$ denotes the diffusion interaction parameter. Characteristic autocorrelation functions from the self-organizing copolymer solution are given in Figure S1 of the SIS.

Furthermore, $k_{D}$ is related to the second virial coefficient $A_{2}$ according to $k_{D}=2A_{2}M-k_{f}-u$, where M is the molecular weight, $k_{f}$ the concentration dependence of the friction coefficient, and u the polymer’s partial specific volume.

SLS data were processed according to the Debye formalism, employing the equation: $\frac{Kc}{\Delta R_{\theta }}=\frac{1}{M_{w}}+2A_{2}c$ where the optical constant K is an optical constant incorporating the refractive index increment dn/dc, and $\Delta R_{\theta }$ represents the excess Rayleigh ratio relative to the solvent. Refractive index increments measurements were performed on a Chromatix KMX-16 refractometer from Milton Roy, operating at 633 nm and calibrated with aqueous NaCl solutions. These analyses enabled the determination of molecular weight and second virial coefficient values.

### 2.3. Transmission Electron Microscopy (TEM) Measurements

Transmission electron microscopy (TEM) experiments were conducted employing a Hitachi model HΤ7800. For the preparation of the samples, carbon-coated copper TEM grids on 200 mesh (ProSciTech Pty. Ltd., Thurnigowa, Australia) were used. An aliquot (5 μL) of the sample was pipetted onto each grid. The solution was allowed to be adsorbed for 2 min. The grids were then manually blotted using Whatman 541 filter paper to remove the excess liquid on the grids. The grids were then left to air-dry at room temperature for approximately 20 min. No staining protocol was applied to the grid preparation for all samples.

### 2.4. Encapsulation of Curcumin and Indomethacin

#### 2.4.1. Preparation of Stock Solutions for Drug Encapsulation

The stock solutions employed for the study of the encapsulation of curcumin and indomethacin within the dry core of the micellar structures formed by the amphiphilic diblock copolymers in water were prepared with the same methodology as those utilized for the DLS and SLS measurements. The only difference is the following: the initial solution of the diblock copolymer, dissolved in THF, was equally distributed between separate vials. Before the addition of only 5 mL of extra pure water on this occasion, a solution of the drug in THF was introduced into these vials at varying drug-to-polymer mass ratios. The vials were then left overnight to ensure full interaction between the drug and the polymer. Subsequently, the remaining steps of the procedure were identical to those previously mentioned.

At this point, it would be beneficial to provide a concrete illustration concerning concentrations. Approximately 20 mg of a sample IS dissolved in 20 mL of dry THF. This solution is equally divided into 5 mL proportions in four vials. To these vials, a solution of indomethacin in THF is introduced at concentrations of 10, 15, and 20% *w*/*w* indomethacin or curcumin/polymer. The solution that remains without the administration of a drug is utilized as a blank for the UV-Vis measurements, as described below. The precise concentrations of polymers and drugs are provided, along with the results obtained in the Results and Discussion section.

#### 2.4.2. Drug Loading Capacity and Efficiency (%DLC and %DLE) Calculation

It has previously been established that both curcumin and indomethacin absorb at 429 and 320 nm, respectively. Consequently, UV-Vis spectroscopy was employed for their quantitative determination in the solutions in which their encapsulation was attempted. The experiments were conducted on a Lamda 650 spectrophotometer from Perkin Elmer, operating within a wavelength range of 250 to 800 nm, at 25 °C, using a 3 mL quartz cell.

Initially, two separate calibration curves were created from UV-Vis spectroscopy, measuring the absorbance of curcumin and indomethacin solutions in THF with varying concentrations. Using the corresponding calibration curve and measuring the absorbance of the polymer solutions with the encapsulated drugs, two critical parameters, namely drug loading capacity (%DLC) and drug loading efficiency (%DLE), were evaluated via the following equations:

(1) $$Drug\;Loading\;Capacity\;\left(DLC\right)\%=\frac{mass\;of\;loaded\;drug}{mass\;of\;polymer}*100$$

(2) $$Drug\;Loading\;Efficiency\;\left(DLE\right)\;\%=\frac{mass\;of\;loaded\;drug}{mass\;of\;drug\;in\;feed}*100$$

## 3. Results and Discussion

### 3.1. Self-Assembly Behavior of the Amphiphilic Diblock Copolymers in Aqueous Solutions

#### 3.1.1. DLS Results for the PNVP-b-PVBu Samples

As illustrated in Table 1, sample PNVP-b-PVBu #2 exhibits the highest percentage of the PNVP block and the lowest percentage of the PVBu block among the other PNVP-b-PVBu samples. This suggests that the micellar structures formed from this sample in water are expected to have a large shell and a small core, thus falling under the classification of the star-like micelles. In contrast, sample PNVP-b-PVBu #1 represents the opposite extreme, containing the highest percentage of the PVBu block and the lowest percentage of the PNVP block. Consequently, the expected morphology of the micellar structures formed from this sample is reversed, featuring a comparatively thin shell and an expanded core, which places it in the category of crew-cut micelles. The remaining three samples have a roughly equivalent PNVP block yet vary from each other in terms of the percentage of their hydrophobic PVBu block.

**Figure 1:**
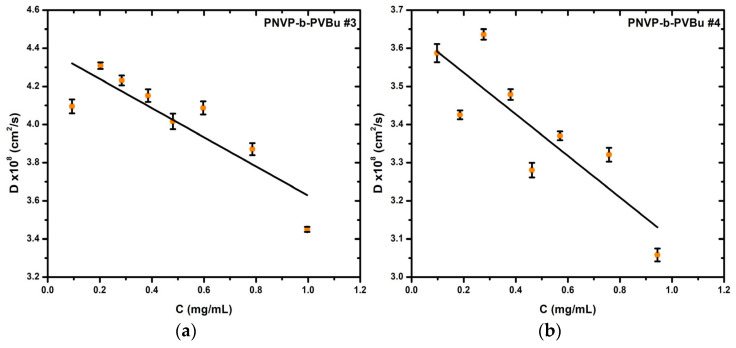
(**a**) Apparent diffusion coefficient vs. concentration plot of sample PNVP-b-PVBu #3; (**b**) Apparent diffusion coefficient vs. concentration plot of sample PNVP-b-PVBu #4.

The experimental results of the DLS analysis for the PNVP-b-PVBu samples are summarized in Table 2, and representative DLS plots are displayed in Figure 1.

The R_h0_ values refer to the hydrodynamic radius at infinite dilution coming from the cumulant analysis and the plots of diffusion coefficient versus concentration. In case of the existence of two populations, the R_h1_ and R_h2_ values refer to the results given by the CONTIN algorithm. Sample PNVP-b-PVBu #1, which contains the highest percentage of the PVBu block, according to both CONTIN analysis and the results demonstrated in Table 2, exhibits only a single population of particles in aqueous solutions with $R_{h0}=92.4$ nm, and a polydispersity index of 0.1<PDI<0.2. This finding suggests that the supramolecular structures formed are relatively uniform, most notably when considering the relatively broad polydispersity, Đ=1.90, of the initial diblock copolymer PNVP-b-PVBu #1. This result, in conjunction with the large size of detected structures, indicates the presence of either micellar aggregates or vesicles.

In contrast with the sample PNVP-b-PVBu #1, the remaining samples with the lower percentage of the hydrophobic PVBu block, as revealed by CONTIN analysis and whose results are presented in Table 2, exhibit two distinct populations with dimensions ranging from 20 to 30 nm and 100 to 130 nm, respectively. Taking into consideration the low molecular weights of the diblock copolymers, it can be concluded that there is an equilibrium between micelles and either micellar aggregates or vesicles. In each sample, the overall PDI attains relatively high values, due to the aforementioned equilibrium; however, each individual population exhibits a notable uniform size, a finding that is in alignment with the observations derived from sample PNVP-b-PVBu #1.

In the case of the sample PNVP-b-PVBu #2, a substantial enhancement in the fraction of micellar aggregates or vesicles was observed, with an increase from 30% at low concentrations up to 55% at high concentrations. Upon increasing concentration, the micellar structures approach one another, thereby facilitating interactions mediated by the hydrophilic PNVP block. This phenomenon may ultimately lead to either the formation of micellar aggregates or to the generation of vesicles, accompanied by rearrangement of the hydrophilic and hydrophobic segments of the copolymers.

In the case of the remaining samples, namely PNVP-b-PVBu #3, #4 and #5, which contain a higher percentage of the hydrophobic PVBu block ranging from 43% to 52%, the predominance of micellar aggregates or vesicles over the simple micellar structures is clearly evident, as their percentage ranges from 65% to 80% according to CONTIN analysis. Additionally, the variation of this fraction with respect to concentration is minimal. The increased percentage of the hydrophobic PVBu block results in more stable and compact micellar cores, and in conjunction with the small PNVP water-soluble block, it facilitates micelle–micelle interactions, ultimately favoring the formation of micellar aggregates or vesicles. The linearity of the plots of diffusion coefficients with concentration (Figure 1) is not perfect, since these points are coming from the cumulant analysis and reflect the equilibrium between micelles and larger supramolecular structures. Upon increasing the concentration, the equilibrium shifts in favor of the larger aggregates.

The $k_{D}$ value for the sample PNVP-b-PVBu #1 is negative, indicating that hydrodynamic interactions are predominant over thermodynamic ones. The negative $k_{D}$ value further suggests that the molecular weight of the supramolecular structures is not particularly high, thereby implying that the degree of aggregation is not very high. This finding aligns more closely with the presence of micellar aggregates or vesicles in solutions, which are defined by an elevated size yet comparatively low molecular weight, as opposed to micellar aggregates.

**Figure 2:**
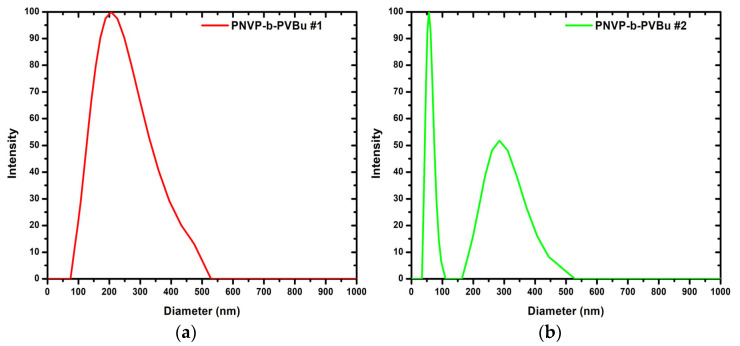
(**a**) CONTIN plot of sample PNVP-b-PVBu #1 at C = 0.59 mg/mL; (**b**) CONTIN plot of sample PNVP-b-PVBu #2 at C = 0.289 mg/mL.

A similar behavior is also observed in the individual distribution profiles corresponding to the two particle populations detected in the other samples by CONTIN analysis. Two characteristic CONTIN plots of sample PNVP-b-PVBu #1, which exhibits one population, and of sample #2 with two populations are given in Figure 2. Additional CONTIN plots are given in Figures S2 and S3 in the Supporting Information Section (SIS).

#### 3.1.2. DLS Results for the PNVP-b-PVDc Samples

**Figure 3:**
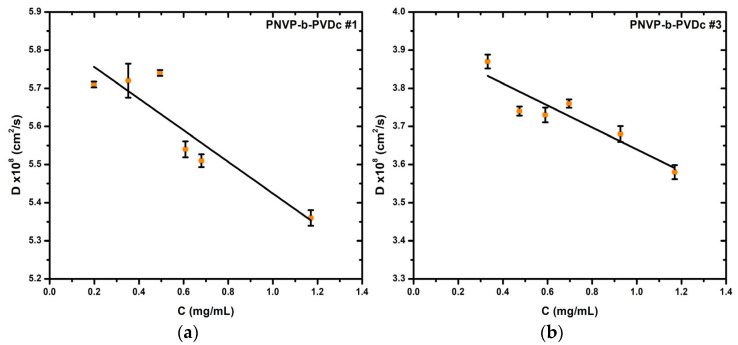
(**a**) Apparent diffusion coefficient vs. concentration plot of sample PNVP-b-PVDc #1; (**b**) apparent diffusion coefficient vs. concentration plot of sample PNVP-b-PVDc #3.

As can be seen from the data in Table 1, all PNVP-b-PVDc samples display remarkably similar molecular weights. Consequently, the results are directly comparable in terms of molecular weight and rely exclusively on the composition of the copolymers. The results of the DLS analysis are summarized in Table 3, and characteristic DLS plots are featured in Figure 3.

The initial observation, which is directly related to the composition of the samples, is that the overall hydrodynamic radii of the supramolecular structures formed in aqueous solution increase with increasing percentage of the hydrophobic PVDc block.

**Figure 4:**
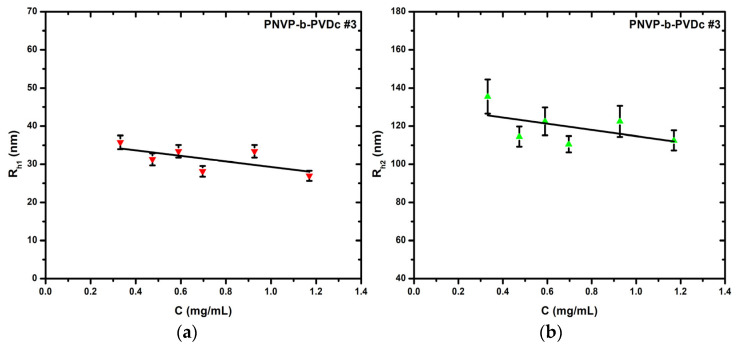
(**a**) R_h1_ vs. C plot of sample PNVP-b-PVDc #3 in H_2_O; (**b**) R_h2_ vs. C plot of sample PNVP-b-PVDc #3 in H_2_O.

**Figure 5:**
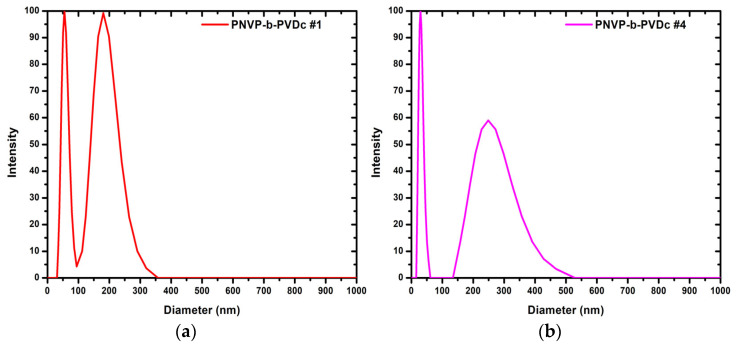
(**a**) CONTIN plot of sample PNVP-b-PVDc #1 at C = 0.352 mg/mL; (**b**) CONTIN plot of sample PNVP-b-PVDc #4 at C = 0.925 mg/mL.

In addition, the CONTIN analysis reveals the presence of two distinct populations, a behavior consistent with that observed for the PNVP-b-PVBu samples, with the exception of PNVP-b-PVBu #1. Plots of the hydrodynamic radius of the two populations are displayed in Figure 4. The linearity of the plots is not perfect, since the system is in dynamic equilibrium, and in addition, the polydispersity of the structures is significant. The relative dimensions of the two populations further indicate that there is an equilibrium between micelles and micellar aggregates or vesicles. In samples PNVP-b-PVDc #1, #2, and #3, the larger structures are predominant. Increasing the concentration of the solutions used for the DLS measurements reduces the contribution of simple micelles and shifts the equilibrium further toward the larger aggregates. The only exception to this is sample PNVP-b-PVDc #4, where the smaller micellar structure prevails, due to the fact that the percentage of the hydrophobic PVDc block in this sample is only 7%, as shown in Table 1. Representative CONTIN analysis plots are given in Figure 5 for the samples PNVP-b-PVDc #1 and #4. The CONTIN plots of the remaining two PNVP-b-PVDc samples are provided in Figure S4 in SIS.

#### 3.1.3. DLS and SLS Results for the PNVP-b-PVSt Samples

**Figure 6:**
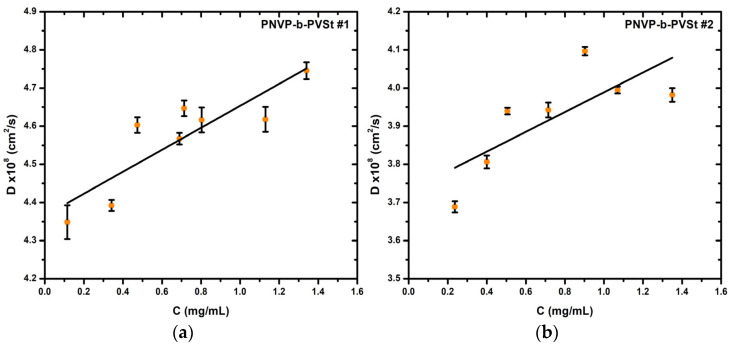
(**a**) Apparent diffusion coefficient vs. concentration plot of sample PNVP-b-PVSt #1; (**b**) apparent diffusion coefficient vs. concentration plot of sample PNVP-b-PVSt #2.

As observed in the case of the amphiphilic block copolymers containing PVDc, in the last family of samples, namely PNVP-b-PVSt, all copolymers exhibit comparable molecular weights. Therefore, the results, presented in Table 4, again depend only on the composition of the copolymers. Representative DLS plots are also provided in Figure 6.

**Figure 7:**
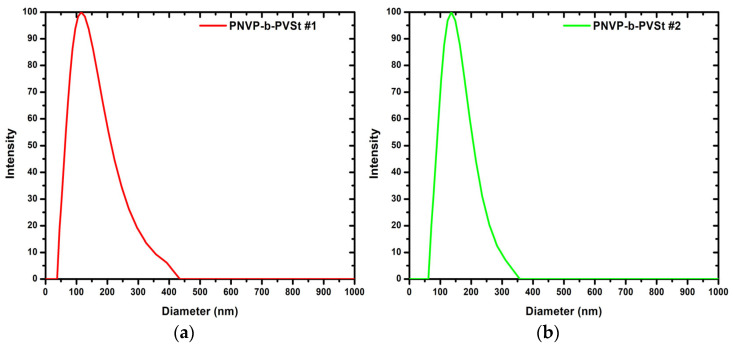
(**a**) CONTIN plot of sample PNVP-b-PVSt #1 at C = 0.115 mg/mL; (**b**) CONTIN plot of sample PNVP-b-PVSt #2 at C = 0.236 mg/mL.

In contrast to all previously discussed systems, and with the sole exception of sample PNVP-b-PVBu #1, CONTIN analysis of the PNVP-b-PVSt samples reveals the presence of a single population across all concentrations of the stock solutions used for the measurements. CONTIN analysis plots are given for the sample PNVP-b-PVSt #1 and PNVP-b-PVSt #2 in Figure 7. The CONTIN analysis plots of samples PNVP-b-PVSt #3, PNVP-b-PVSt #4 and PNVP-b-PVSt #5 are included in SIS in Figures S5 and S6. Taking into account the measured hydrodynamic radii (Table 4) and the relatively low molecular weights of the free chains (Table 1), it can be concluded that the structures formed in aqueous environment correspond either to micelles with a high degree of aggregation, to micellar aggregates, or to stable vesicular assemblies.

In accordance with the behavior previously observed in the PVBu- and PVDc-based amphiphilic block copolymers, the hydrodynamic radius of the supramolecular structures formed in PNVP-b-PVSt samples increases upon increasing the percentage of the hydrophobic PVSt block, most likely due to a higher degree of aggregation.

The values of the PDI for all samples exceed 0.2, indicating that the supramolecular structures formed in aqueous solution exhibit a broad size distribution. It is evident that this phenomenon can be attributed, in part, to the polydispersity of the initial copolymers. However, it is primarily attributable to the aggregation process itself. More specifically, the hydrophobic interactions within the core-forming material in the PNVP-b-PVSt copolymers are stronger compared to the two other families of copolymers. The long alkyl side chains of PVSt are able to form crystalline domains, leading to the formation of compact micellar cores.

In all PNVP-b-PVSt samples, the $k_{D}$ values are found to be relatively low but positive due to the huge M_w_ of the aggregates, thus indicating that thermodynamic interactions predominate over hydrodynamic ones. As a direct consequence, and due to the reduced interactions of the polymeric system with the solvent, the corresponding $A_{2}$ values persist at sufficiently low levels.

**Figure 8:**
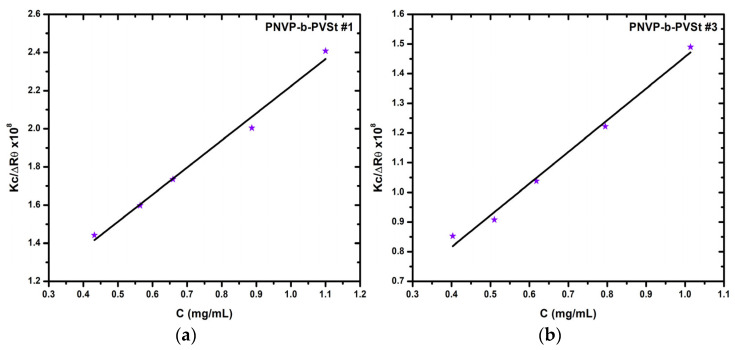
(**a**) SLS plot of sample PNVP-b-PVSt #1; (**b**) SLS plot of sample PNVP-b-PVSt #3.

As already mentioned, the PNVP-b-PVSt samples are the only ones that consistently exhibit a single population. Therefore, SLS measurements for these samples were carried out as well. The detailed results are presented in Table 5, which, for convenience, also includes the information from Table 1 regarding the molar ratio of the hydrophobic PVSt block. Furthermore, characteristic SLS plots are presented in Figure 8.

In agreement with the DLS results, the structures formed upon self-assembly of these amphiphilic block copolymers in aqueous solution are micelles characterized by an exceptionally high degree of aggregation. In general, the degree of aggregation was governed by both the block copolymer composition and the degree of polymerization. Since all samples share nearly identical molecular characteristics, the degree of association is dictated exclusively by the copolymer composition.

More specifically, as the percentage of the hydrophilic PNVP block decreases, corresponding to an increase in the percentage of the hydrophobic PVSt block, the degree of association value, Nw, rises dramatically. For instance, in PNVP-b-PVSt samples #1, #2, and #3, where the PNVP content ranges between 78 and 85%, Nw is already very high, from approximately 3800 up to 5500. Upon reduction in the percentage of the PNVP block to 61% in sample #4, Nw increases to about 11,000, and in sample #5, containing only 40% PNVP, Nw reaches an exceptionally high value of over 48,000. This behavior reflects the formation of a highly compact and rigid hydrophobic core, effectively minimizing the unfavorable hydrophobic–water interfacial area.

### 3.2. Self-Assembly Behaviour of the PNVP-b-PVEs Block Copolymers in Aqueous Solutions by TEM Imaging

**Figure 9:**
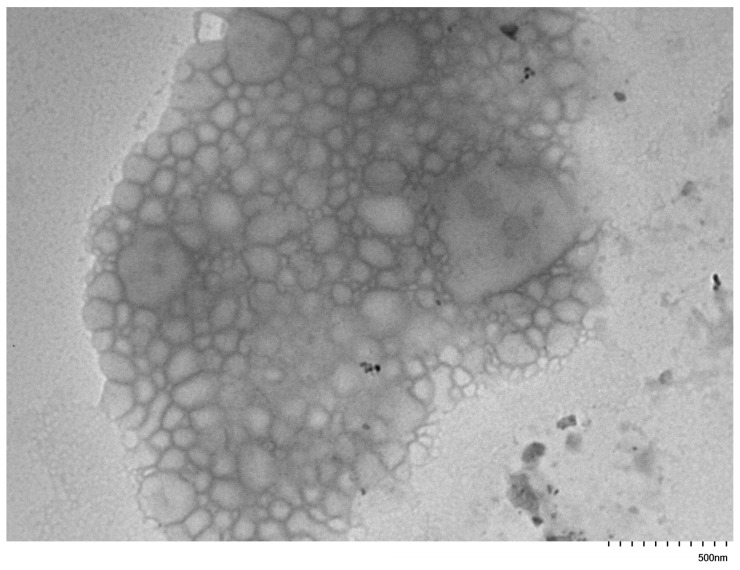
TEM image from the sample PNVP-b-PVBu #1.

**Figure 10:**
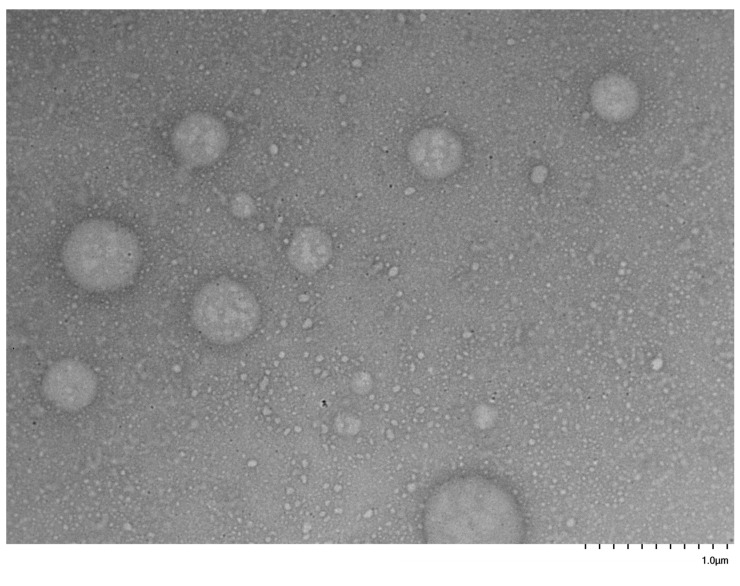
TEM image from the sample PNVP-b-PVBu #2.

**Figure 11:**
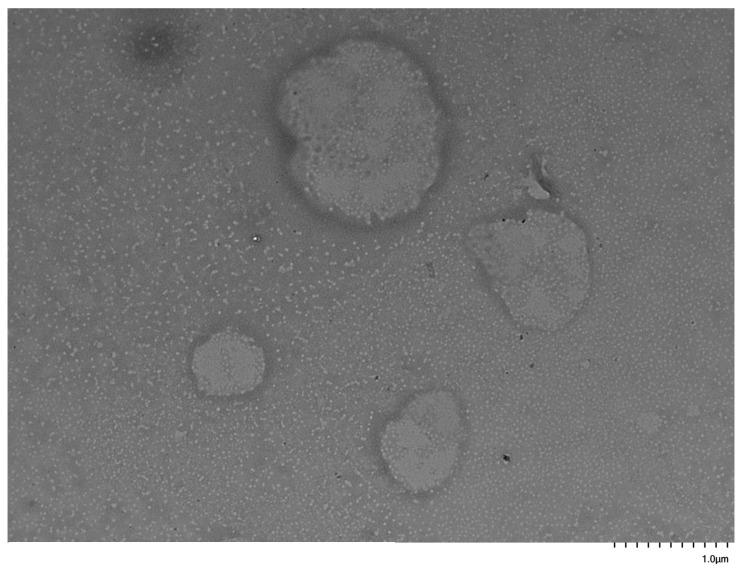
TEM image from the sample PNVP-b-PVDc#1.

**Figure 12:**
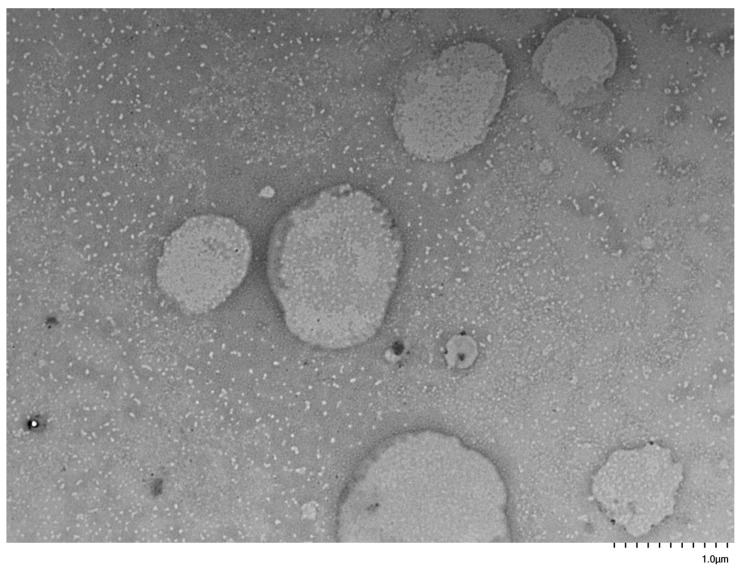
TEM image from the sample PNVP-b-PVDc#4.

**Figure 13:**
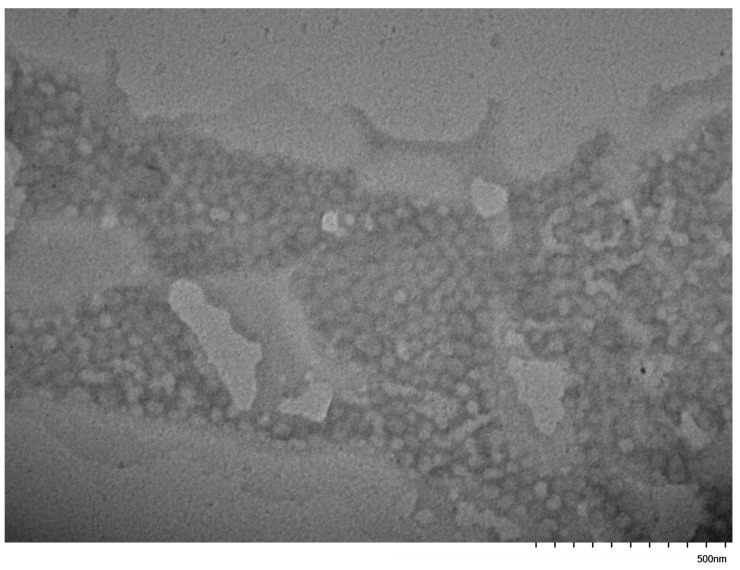
TEM image from the sample PNVP-b-PVSt#3.

**Figure 14:**
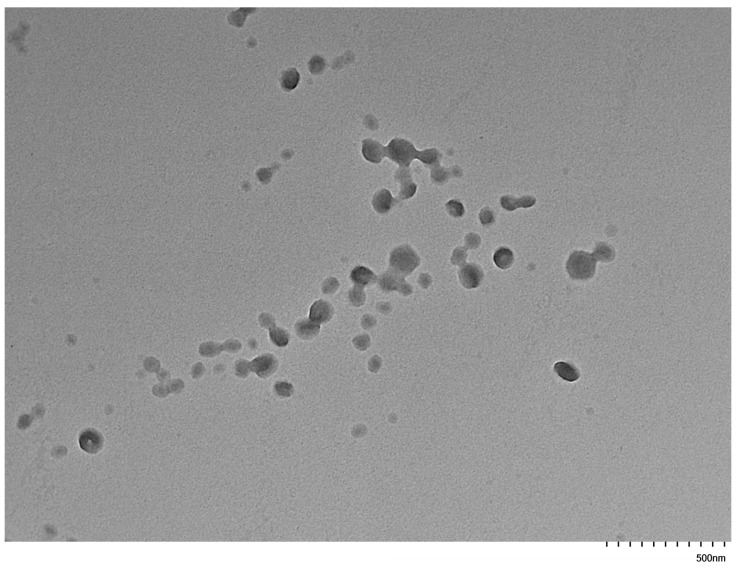
TEM image from the sample PNVP-b-PVSt#5.

TEM images were collected on carbon-coated copper grids. Efforts were made to stain the specimens with uranyl acetate to improve the contrast of the images. However, this procedure was not successful, since uranium strongly interacted through complexation with the nitrogen atoms of the pyrrolidone rings, producing a black surface without any possibility of observing any micellar structure on the grids. Therefore, the images were obtained without staining. Characteristic images from the micellar solutions in THF are given in Figure 9, Figure 10, Figure 11, Figure 12, Figure 13 and Figure 14.

A characteristic image from sample PNVP-b-PVBu#1 is given in Figure 9. As was revealed from the DLS measurements, single micellar, relatively spherical structures were revealed by TEM imaging. In addition, the distribution of sizes is not very broad in agreement with CONTIN analysis. It is characteristic that the size of the micellar structures is lower than that measured by DLS, although in most cases the opposite behaviour is obtained [43,44], due to the expansion of the structure on the surface of the grid in the absence of any solvent. This result is associated with the hydrophilic character of the PNVP chains, which is the corona-forming block, and the hydrophobic character of the carbon-coated copper grids. In this case, the PNVP chains shrink to avoid contact with the hydrophobic surface.

The DLS measurements were also confirmed with sample PNVP-b-PVBu#2. A characteristic image is given in Figure 10. It is clear that in this case, there is an equilibrium between large supramolecular structures with small micelles. The large structures are polydisperse and seem to be constructed as assemblies of multimolecular micelles. Therefore, the scenario of the existence of vesicles from the DLS measurements seems to be abandoned in favour of the presence of micellar aggregates.

Similar conclusions were also drawn for samples PNVP-b-PVDc#1 and #4, as shown by the images given in Figure 11 and Figure 12, respectively.

Finally, for samples PNVP-b-PVSt#3 and #5, the DLS conclusion for the presence of single populations of micelles is confirmed, as shown in Figure 13 and Figure 14, respectively.

### 3.3. Drug Encapsulation Results

#### 3.3.1. Encapsulation of Curcumin

The results of curcumin encapsulation within the dry cores of the micellar structures formed by the self-assembly of the PNVP-b-PVBu amphiphilic block copolymers in aqueous solution are summarized in Table 6. UV-Vis spectra of PNVP-b-PVBu samples with all concentrations of encapsulated curcumin are given in Figures S7 and S8 in SIS.

As demonstrated in Table 6, the encapsulation efficiency is found to be noteworthy, with the DLE values attaining a maximum of 80%. As might have been anticipated, sample PNVP-b-PVBu #1, which contains the highest amount of the hydrophobic PVBu block and exhibits a single population of micellar aggregates, as evidenced by the DLS results in Table 2, shows the highest encapsulation capacity.

Among the three remaining samples, PNVP-b-PVBu #2 may demonstrate a slightly higher encapsulation performance due to the larger hydrodynamic radius of its second micellar population; however, this sample, along with samples PNVP-b-PVBu #3 and #4, displays a very similar behavior overall. This observation is consistent with the DLS results presented in Table 2, given the coexistence of micelles with clusters or micellar aggregates in all three cases, as previously discussed.

Overall, the observed behavior is in accordance with the anticipated trend, i.e., the percentage of curcumin encapsulation, increases as the feed concentration increases, as demonstrated by the increase in DLC values. However, in the case of DLE, after an initial rise, DLE values appear to level off, reaching a plateau with nearly constant values.

The corresponding results for the PNVP-b-PVDc samples are presented in Table 7, and the respective UV-Vis spectra are featured in Figures S9 and S10 in SIS. The values of DLC and DLE are satisfactory and quite comparable to those of the PNVP-b-PVBu samples, which is consistent with the similar core sizes of the micellar structures formed through their self-assembly in aqueous environments, as also indicated by the hydrodynamic radii of the second micellar populations, summarized in Table 2 and Table 3, for the PNVP-b-PVBu samples and the PNVP-b-PVDc samples, respectively. Nevertheless, the increase in the hydrophobic segment length of the n-alkyl side chain in PVDc compared to that of PVBu results in a rise in the DLE values, most likely due to stronger interactions with curcumin.

More specifically, in the case of the PNVP-b-PVDc amphiphilic block copolymers, for samples #2 and #3, the DLE values increase with increasing concentration of curcumin in the feed. On the other hand, for samples #3 and #4, as the concentration of curcumin in the feed increases from C_3_ to C_4_, a slight decrease in DLE values is observed.

This phenomenon was also noted to a smaller extent in the PNVP-b-PVBu samples and may be attributed to the fact that at higher concentrations, curcumin tends to form aggregates and precipitate. Such behavior is not uncommon for organic dyes, where molecular aggregation in solution frequently occurs, notably in the form of H-aggregates and J-aggregates, particularly at elevated dye concentrations [45,46,47,48]. It is characteristic that dye aggregates do not exhibit the same absorption maximum wavelength as the individual dye molecules, due to slight modifications of the conjugated double-bond system within the aggregate compared to the isolated chromophore. Consequently, in some cases, anomalous absorption behavior is observed in the visible region as a function of the dye concentration in the measured solution.

The investigation of curcumin encapsulation within the micellar structures of the PNVP-b-PVSt amphiphilic block copolymer samples yielded promising results, as summarized in Table 8. The characteristic UV-Vis spectra are all displayed in Figures S11 and S12 in SIS. The hydrophobic drug was successfully incorporated to a satisfactory extent, with all samples exhibiting DLE values above 40%. Similarly to the case of the PNVP-b-PVBu samples, the DLE initially tends to increase until it reaches a plateau with nearly constant values. However, for the PNVP-b-PVSt samples, there is a more specific explanation. As previously mentioned, the long alkyl side chains of PVSt are capable of forming crystalline domains [49], which reduce the capacity of the compact core, thereby limiting the available space for the encapsulation of higher amounts of curcumin.

#### 3.3.2. Encapsulation of Indomethacin

**Figure 15:**
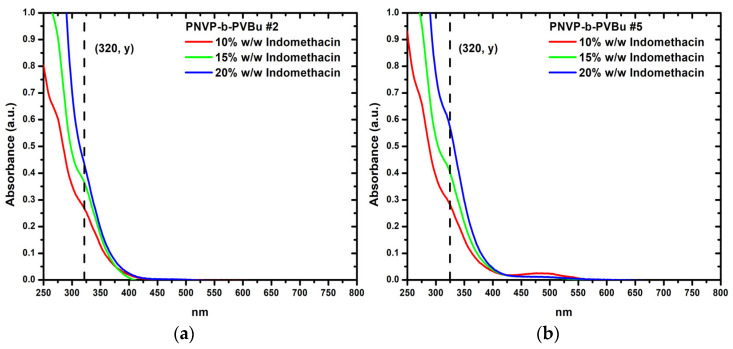
(**a**) UV-Vis spectra of PNVP-b-PVBu #2 with all concentrations of encapsulated indomethacin; (**b**) UV-Vis spectra of PNVP-b-PVBu #5 with all concentrations of encapsulated indomethacin.

In addition to the encapsulation of curcumin, the encapsulation of the hydrophobic drug indomethacin within the dry cores of the micellar structures formed by the self-assembly of the PNVP-b-PVEs amphiphilic block copolymers in aqueous solution was also investigated. The results of the encapsulation of indomethacin into the PNVP-b-PVBu samples are presented in Table 9. As mentioned, UV-Vis spectroscopy was utilized for the quantitative determination of indomethacin in the solutions in which its encapsulation was attempted; therefore, the UV-Vis spectra of indomethacin with various concentrations and the calibration curve for indomethacin at 320 nm are given in SIS Figure S13. The UV-Vis spectra of sample PNVP-b-PVBu #2 and PNVP-b-PVBu #5, respectively, with all three different concentrations of encapsulated indomethacin combined, are provided in Figure 15, while the UV-Vis spectra of the remaining PNVP-b-PVBu samples are included in Figures S14 and S15 in SIS.

Sample PNVP-b-PVBu #1, which, as shown in its composition in Table 1, contains the highest percentage of the hydrophobic PVBu block, displays the highest DLC and DLE values for the encapsulation of indomethacin, consistent with the trend previously observed for curcumin. Remarkably, the addition of up to 20% *w*/*w* indomethacin leads to nearly quantitative encapsulation. In contrast to sample #1, PNVP-b-PVBu #2 shows the lowest DLC and DLE values, precisely because, as indicated in Table 1, it contains the lowest percentage of the hydrophobic PVBu block.

The three remaining samples, PNVP-b-PVBu #3, #4 and #5, which possess similar compositions and intermediate percentages of the hydrophobic PVBu block relative to the two extreme cases of samples #1 and #2, exhibit comparable behaviors and DLC/DLE values, falling between those observed for samples #1 and #2, as expected.

**Figure 16:**
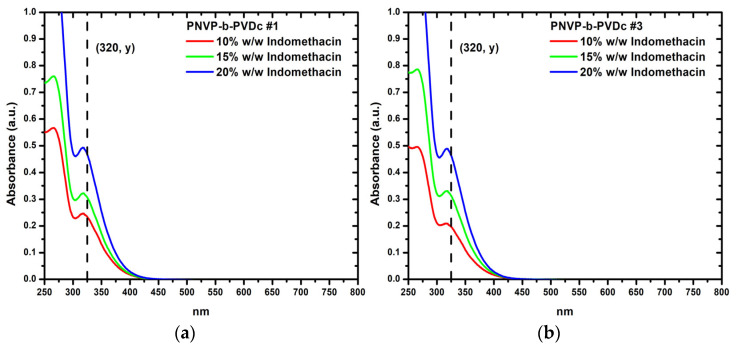
(**a**) UV-Vis spectra of PNVP-b-PVDc #1 with all concentrations of encapsulated indomethacin (**b**) UV-Vis spectra of PNVP-b-PVDc #3 with all concentrations of encapsulated indomethacin.

The results regarding the encapsulation of indomethacin within the PNVP-b-PVDc samples are summarized in Table 10, and characteristic UV-Vis spectra are given in Figure 16. Additional UV-Vis spectra are provided in Figure S16 in the SIS.

The encapsulation results of indomethacin in the PNVP-b-PVDc samples are remarkably high. The DLC values closely match the respective feed concentrations of indomethacin, while the DLE demonstrates that in the vast majority of samples, the incorporation of indomethacin into the dry micellar core is essentially quantitative. These findings highlight that indomethacin is more efficiently accommodated within the micelles compared to curcumin. In the case of PNVP-b-PVDc, the encapsulation efficiency surpasses that of the PNVP-b-PVBu samples, which can be attributed to the longer n-alkyl hydrophobic side chains of PVDc that promote stronger hydrophobic interactions with the hydrophobic drug molecules.

**Figure 17:**
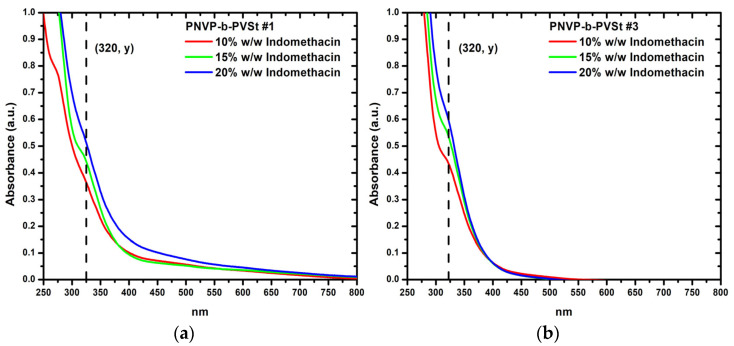
(**a**) UV-Vis spectra of PNVP-b-PVSt #1 with all concentrations of encapsulated indomethacin (**b**) UV-Vis spectra of PNVP-b-PVSt #3 with all concentrations of encapsulated indomethacin.

Finally, the encapsulation performance of indomethacin within the PNVP-b-PVSt samples is highlighted in Table 11, accompanied by UV-Vis spectra in Figure 17. Additional UV-Vis spectra are given in Figures S17 and S18 in the SIS.

All PNVP-b-PVSt samples exhibit consistent behavior. At an indomethacin feed concentration of 10% *w*/*w* indomethacin/polymer, encapsulation is nearly quantitative, with DLE values approaching 100%. However, further increases in feed concentration lead to a sharp decline in DLE values. This behavior contrasts with that of PNVP-b-PVBu and PNVP-b-PVDc, yet it can be fully explained by the nature of the micelles. In the PNVP-b-PVDc samples, longer n-alkyl side chains enhance loading capacity and efficiency, compared to PNVP-b-PVBu samples, due to the amorphous micellar core, which provides ample space for drug incorporation. In PNVP-b-PVSt samples; however, the PVSt block crystallizes, significantly reducing the available core volume. Consequently, full encapsulation is achieved only up to 10% *w*/*w*, corresponding to the observed DLC values, while beyond this threshold, the core becomes saturated, preventing further indomethacin loading, which is clearly reflected in the steep drop of DLE at higher feed concentrations.

## 4. Conclusions

Water acts as a selective solvent for the PNVP-b-PVEs amphiphilic block copolymers, enabling their self-assembly into micellar structures upon dissolution. In the cases of PNVP-b-PVBu and PNVP-b-PVDc copolymers, where $k_{D}$ values are negative, interparticle hydrodynamic interactions prevail, whereas in the case of PNVP-b-PVSt samples, self-assembly in aqueous media is a thermodynamically driven process, as indicated by the positive $k_{D}$ values. The investigation of the self-assembly of PNVP-b-PVEs, which differ in the length of their n- alkyl side chains, further demonstrated the impact of this parameter on their micellization behavior. Specifically, PNVP-b-PVBu and PNVP-b-PVDc samples, bearing short- and medium-length side chains, respectively, exhibited an equilibrium between two populations of micelles and micelle aggregates, whereas in the PNVP-b-PVSt samples, with the longest aliphatic side chain, only a single population was detected. Within each copolymer family, the effect of composition on the morphology of the formed structures was also evident. In all cases, increasing the percentage of the hydrophobic PVE block resulted in an enlargement of the micellar core size and, consequently, an increase in the hydrodynamic radius. Encapsulation of hydrophobic bioactive molecules within the micellar cores was successful for both curcumin and indomethacin. These amphiphilic block copolymers proved more effective in encapsulating indomethacin compared to curcumin. Copolymer composition was identified as the key parameter for enhancing loading capacity and efficiency, as higher hydrophobic PVE percentage consistently led to increased DLC and DLE values due to enlargement of the hydrodynamic radius. Comparison among the three PNVP-b-PVEs copolymer families revealed that with PVEs having short alkyl side chains, the DLC and DLE values either increase or at least remain stable upon increasing the amount of the drug, whereas with PVEs with long alkyl side chains, DLC and DLE decrease in the same sense. This is due to the stronger interaction of the long side chains and the ability to form crystalline domains that restrict the incorporation of higher amounts of drug. In summary, the morphology, size, and stability of the self-assembled nanostructures, combined with the rather high DLC and DLE values, particularly in the case of indomethacin, strongly suggest that the amphiphilic block copolymers investigated in this study can be considered as possible carriers for drug delivery applications.

## Figures and Tables

**Table 1:** Molecular characteristics of the PNVP-b-PVEs amphiphilic diblock copolymers.

Sample	PNVP ^1^		PNVP-b-PVEs ^1^		NVP ^2^	PVEs ^2^
	M_n_ ×10^3^ (Da)	Đ	M_n_ ×10^3^ (Da)	Đ	Molar Ratio (%)	Molar Ratio (%)
PNVP-b-PVBu #1	8.5	1.30	16.0	1.90	22	78
PNVP-b-PVBu #2	28.0	1.27	32.0	1.32	84	16
PNVP-b-PVBu #3	8.9	1.35	17.5	1.40	57	43
PNVP-b-PVBu #4	8.9	1.35	15.5	1.54	48	52
PNVP-b-PVBu #5	8.5	1.30	13.0	1.55	56	44
PNVP-b-PVDc #1	8.5	1.30	12.5	1.31	63	37
PNVP-b-PVDc #2	5.5	1.47	12.5	1.60	38	62
PNVP-b-PVDc #3	8.5	1.30	11.0	1.45	56	44
PNVP-b-PVDc #4	9.5	1.36	10.5	1.36	93	7
PNVP-b-PVSt #1	8.5	1.30	10.5	1.44	78	22
PNVP-b-PVSt #2	7.5	1.30	10.4	1.51	61	39
PNVP-b-PVSt #3	8.1	1.30	10.9	1.37	85	15
PNVP-b-PVSt #4	8.1	1.30	12.5	1.22	83	17
PNVP-b-PVSt #5	5.5	1.47	9.0	1.62	40	60

^1^ Derived by size exclusion chromatography (SEC) in CHCl_3_. ^2^ Derived by ^1^H-NMR in CDCl_3_.

**Table 2:** DLS results for the PNVP-b-PVBu samples.

Sample	D_0_ ×10^8^ (cm^2^/s)	K_D_	R_h0_ (nm)	R_h1,0_ (nm)	R_h2,0_ (nm)
PNVP-b-PVBu #1	2.653	−86.11	92.4	-	-
PNVP-b-PVBu #2	4.813	177.46	50.9	30.8	127.6
PNVP-b-PVBu #3	4.392	−174.02	55.8	23.1	104.6
PNVP-b-PVBu #4	3.645	−149.42	67.3	29.5	99.2
PNVP-b-PVBu #5	6.198	362.45	39.6	20.3	115.7

**Table 3:** DLS results for the PNVP-b-PVDc samples.

Sample	D_0_ ×10^8^ (cm^2^/s)	K_D_	R_h0_ (nm)	R_h1,0_ (nm)	R_h2,0_ (nm)
PNVP-b-PVDc #1	5.84	−71	42.0	24.2	91.8
PNVP-b-PVDc #2	2.88	−502	85.0	15.6	95.4
PNVP-b-PVDc #3	3.93	−73	62.4	35.6	131.1
PNVP-b-PVDc #4	6.62	981	37.0	15.4	136.4

**Table 4:** DLS results for the PNVP-b-PVSt samples.

Sample	D_0_ ×10^8^ (cm^2^/s)	K_D_	R_h0_ (nm)
PNVP-b-PVSt #1	4.37	65.8	56.1
PNVP-b-PVSt #2	3.73	69.3	65.7
PNVP-b-PVSt #3	4.52	693.0	54.2
PNVP-b-PVSt #4	4.35	187.8	56.4
PNVP-b-PVSt #5	2.54	69.0	96.6

**Table 5:** SLS results for the PNVP-b-PVSt samples.

Sample	PVSt ^1^ Molar Ratio (%)	(M_w_)_micc_ ^2^ (Daltons)	(A_2_)_micc_ ^2^ (cm^3^mol/g^2^)	Nw
PNVP-b-PVSt #1	22	4.02 × 10^7^	2.15 × 10^−5^	3828
PNVP-b-PVSt #2	39	1.18 × 10^8^	1.65 × 10^−5^	11,346
PNVP-b-PVSt #3	15	5.98 × 10^7^	1.85 × 10^−5^	5486
PNVP-b-PVSt #4	17	4.77 × 10^7^	1.64 × 10^−5^	3816
PNVP-b-PVSt #5	60	4.35 × 10^8^	1.87 × 10^−6^	48,333

^1^ Derived by ^1^H-NMR in CDCl_3_. ^2^ Derived by SLS.

**Table 6:** Results of curcumin encapsulation within the dry core of the micellar structures formed in aqueous solutions by the PNVP-b-PVBu samples.

Sample	Cur. ^1^	A ^2^ (a.u.)	Pol. ^3^ (μg/mL)	DIF ^4^ (μg/mL)	LD ^5^ (μg/mL)	DLC (%)	DLE (%)
PNVP-b-PVBu #1	1%	1.85	481.20	12.60	9.19	1.91	73.01
	2%	1.78	670.09	14.59	8.83	1.32	60.54
	3%	2.85	653.62	17.93	14.33	2.19	79.90
	4%	2.74	643.69	21.22	13.76	2.14	64.85
PNVP-b-PVBu #2	1%	0.45	543.50	6.23	2.00	0.37	32.08
	2%	1.57	604.45	14.17	7.75	1.28	54.72
	3%	1.85	599.34	17.42	9.19	1.53	52.75
	4%	2.55	621.73	21.75	12.79	2.06	58.80
PNVP-b-PVBu #3	1%	0.38	583.23	6.33	1.64	0.28	25.90
	2%	1.84	650.11	14.12	9.14	1.41	64.73
	3%	1.97	637.42	17.22	9.81	1.54	56.94
	4%	2.34	630.40	21.68	11.71	1.86	54.01
PNVP-b-PVBu #4	1%	0.37	537.80	5.97	1.59	0.30	26.61
	2%	1.94	632.52	14.38	9.65	1.53	67.13
	3%	1.93	619.94	17.38	9.60	1.55	55.23
	4%	2.89	638.81	22.02	14.53	2.27	65.99

^1^ Curcumin concentration. ^2^ Absorbance. ^3^ Concentration of the polymer stock solutions in H_2_O. ^4^ Concentration of the drug in feed. ^5^ Concentration of the loaded drug.

**Table 7:** Results of curcumin encapsulation within the dry core of the micellar structures formed in aqueous solutions by the PNVP-b-PVDc samples.

Sample	Cur. ^1^	A ^2^ (a.u.)	Pol. ^3^ (μg/mL)	DIF ^4^ (μg/mL)	LD ^5^ (μg/mL)	DLC (%)	DLE (%)
PNVP-b-PVDc #1	1%	0.61	684.13	6.18	4.82	0.71	78.05
	2%	2.12	746.70	13.79	2.82	0.38	20.46
	3%	2.64	765.59	17.73	10.58	1.38	59.64
	4%	1.92	767.62	21.11	13.25	1.73	62.77
PNVP-b-PVDc #2	1%	0.73	601.64	6.37	9.55	1.59	49.88
	2%	2.17	668.63	13.92	3.44	0.51	24.69
	3%	2.68	676.55	17.69	10.83	1.60	61.25
	4%	1.69	641.88	20.09	13.45	2.10	66.96
PNVP-b-PVDc #3	1%	0.93	615.39	6.91	8.37	1.36	21.18
	2%	2.08	619.55	14.25	4.46	0.72	31.33
	3%	2.52	592.96	17.34	10.37	1.75	59.81
	4%	2.19	624.96	21.81	12.63	2.02	57.93
PNVP-b-PVDc #4	1%	0.86	560.35	66.07	10.94	1.95	65.54
	2%	1.96	552.45	13.03	4.11	0.74	31.50
	3%	2.21	546.12	16.24	9.76	1.79	60.06
	4%	2.81	544.62	19.41	11.04	2.03	56.87

^1^ Curcumin concentration. ^2^ Absorbance. ^3^ Concentration of the polymer stock solutions in H_2_O. ^4^ Concentration of the drug in feed. ^5^ Concentration of the loaded drug.

**Table 8:** Results of curcumin encapsulation within the dry core of the micellar structures formed in aqueous solutions by the PNVP-b-PVSt samples.

Sample	Cur. ^1^	A ^2^ (a.u.)	Pol. ^3^ (μg/mL)	DIF ^4^ (μg/mL)	LD ^5^ (μg/mL)	DLC (%)	DLE (%)
PNVP-b-PVSt #1	1%	0.63	553.69	6.20	2.92	0.53	47.17
	2%	1.48	570.94	13.26	7.29	1.28	54.98
	3%	1.61	522.77	14.91	7.96	1.52	53.36
	4%	2.24	567.39	19.22	11.19	1.97	58.25
PNVP-b-PVSt #2	1%	0.67	567.63	6.25	3.13	0.55	50.04
	2%	1.74	571.86	12.87	8.63	1.51	67.01
	3%	1.95	580.85	16.02	9.70	1.67	60.59
	4%	2.38	577.08	19.90	11.91	2.06	59.87
PNVP-b-PVSt #3	1%	0.68	554.27	6.15	3.18	0.57	51.70
	2%	1.57	574.55	12.91	7.75	1.35	60.06
	3%	1.86	574.47	16.01	9.24	1.61	57.74
	4%	2.51	591.43	19.06	12.58	2.13	65.99
PNVP-b-PVSt #4	1%	0.48	554.60	5.42	2.15	0.39	39.72
	2%	1.48	560.06	11.74	7.29	1.30	62.12
	3%	2.04	558.56	14.81	10.17	1.82	68.64
	4%	2.19	566.32	18.06	10.94	1.93	60.55

^1^ Curcumin concentration. ^2^ Absorbance. ^3^ Concentration of the polymer stock solutions in H_2_O. ^4^ Concentration of the drug in feed. ^5^ Concentration of the loaded drug.

**Table 9:** Results of indomethacin encapsulation within the dry core of the micellar structures formed in aqueous solutions by the PNVP-b-PVBu samples.

Sample	Ind. ^1^ (*w*/*w*)	A ^2^ (a.u.)	Pol. ^3^ (μg/mL)	DIF ^4^ (μg/mL)	LD ^5^ (μg/mL)	DLC (%)	DLE (%)
PNVP-b-PVBu #1	10%	0.32	178.34	17.16	12.53	7.02	73.00
	15%	0.69	169.51	24.48	28.93	17.07	118.17
	20%	0.80	168.85	34.39	33.98	20.13	98.83
PNVP-b-PVBu #2	10%	0.27	168.70	15.97	10.56	6.26	66.16
	15%	0.37	164.15	25.65	14.93	9.09	58.21
	20%	0.45	166.63	33.01	18.24	10.95	55.26
PNVP-b-PVBu #3	10%	0.32	180.86	16.18	12.55	6.94	77.58
	15%	0.40	182.12	25.69	16.12	8.85	62.76
	20%	0.61	187.77	34.82	25.70	13.69	73.82
PNVP-b-PVBu #4	10%	0.30	171.48	16.54	11.90	6.94	71.95
	15%	0.32	173.91	23.46	42.45	7.16	53.07
	20%	0.56	173.42	32.31	23.32	13.45	72.18
PNVP-b-PVBu #5	10%	0.30	165.14	15.93	11.75	7.11	73.73
	15%	0.43	165.03	24.82	17.41	10.55	70.13
	20%	0.61	164.20	35.00	25.46	15.51	72.75

^1^ Concentration of the indomethacin solutions in THF (*w*/*w* indomethacin/polymer). ^2^ Absorbance. ^3^ Concentration of the polymer stock solutions in H_2_O. ^4^ Concentration of the drug in feed. ^5^ Concentration of the loaded drug.

**Table 10:** Results of indomethacin encapsulation within the dry core of the micellar structures formed in aqueous solutions by the PNVP-b-PVDc samples.

Sample	Ind. ^1^ (*w*/*w*)	A ^2^ (a.u.)	Pol. ^3^ (μg/mL)	DIF ^4^ (μg/mL)	LD ^5^ (μg/mL)	DLC (%)	DLE (%)
PNVP-b-PVDc #1	10%	0.24	73.25	17.63	9.18	12.53	52.05
	15%	0.32	94.67	16.09	12.57	13.28	78.09
	20%	0.49	98.63	19.59	20.06	20.34	102.38
PNVP-b-PVDc #2	10%	0.39	95.43	10.15	15.66	16.41	154.22
	15%	0.55	98.59	15.65	22.65	22.98	144.74
	20%	0.69	101.96	19.60	29.14	28.58	148.66
PNVP-b-PVDc #3	10%	0.20	107.78	10.36	7.46	6.92	72.02
	15%	0.33	109.01	17.25	12.88	11.82	74.97
	20%	0.48	105.61	19.48	19.90	18.84	102.16
PNVP-b-PVDc #4	10%	0.26	97.04	10.24	10.00	10.31	97.64
	15%	0.31	94.03	16.07	11.98	12.75	74.59
	20%	0.52	92.95	19.92	21.67	23.32	108.77

^1^ Concentration of the indomethacin solutions in THF (*w*/*w* indomethacin/polymer). ^2^ Absorbance. ^3^ Concentration of the polymer stock solutions in H_2_O. ^4^ Concentration of the drug in feed. ^5^ Concentration of the loaded drug.

**Table 11:** Results of indomethacin encapsulation within the dry core of the micellar structures formed in aqueous solutions by the PNVP-b-PVSt samples.

Sample	Ind. ^1^ (*w*/*w*)	A ^2^ (a.u.)	Pol. ^3^ (μg/mL)	DIF ^4^ (μg/mL)	LD ^5^ (μg/mL)	DLC (%)	DLE (%)
PNVP-b-PVSt #1	10%	0.39	165.90	15.95	15.66	9.44	98.19
	15%	0.46	164.14	23.65	18.75	11.42	79.26
	20%	0.55	166.71	38.53	22.68	13.60	58.87
PNVP-b-PVSt #2	10%	0.73	32.93	3.27	31.06	9.43	94.9
	15%	0.36	166.27	24.02	14.17	8.52	58.98
	20%	0.65	166.17	33.13	27.49	16.54	82.98
PNVP-b-PVSt #3	10%	0.45	165.82	18.98	18.22	10.99	96.01
	15%	0.55	181.78	27.56	22.82	12.55	82.80
	20%	0.62	168.32	33.06	25.75	15.30	77.90
PNVP-b-PVSt #4	10%	0.41	172.92	16.89	16.71	9.66	98.93
	15%	0.51	167.90	24.12	21.07	12.55	87.33
	20%	0.55	171.99	35.92	22.74	13.22	63.30
PNVP-b-PVSt #5	10%	0.84	157.27	16.47	35.96	22.86	218.31
	15%	0.84	152.17	25.01	35.64	23.42	142.47
	20%	0.90	150.52	33.06	38.35	25.48	116.01

^1^ Concentration of the indomethacin solutions in THF (*w*/*w* indomethacin/polymer). ^2^ Absorbance. ^3^ Concentration of the polymer stock solutions in H_2_O. ^4^ Concentration of the drug in feed. ^5^ Concentration of the loaded drug.

## Data Availability

The original contributions presented in this study are included in the article/Supplementary Materials. Further inquiries can be directed to the corresponding author.

## Supplementary Materials

The following supporting information can be downloaded at: https://www.mdpi.com/article/10.3390/polym17212852/s1, Supplementary Material File S1. Synthesis of the PNVP-b-PVEs block copolymers. Figure S1. Autocorrelation functions from DLS experiments from various block copolymer micellar aqueous solutions. Figure S2. (a) CONTIN plot of sample PNVP-b-PVBu #3 at C = 0.093 mg/mL; (b) CONTIN plot of sample PNVP-b-PVBu #4 at C = 0.758 mg/mL; Figure S3: CONTIN plot of sample PNVP-b-PVBu #5 at C = 0.095 mg/mL; Figure S4: (a) CONTIN plot of sample PNVP-b-PVDc #2 at C = 0.659 mg/mL; (b) CONTIN plot of sample PNVP-b-PVDc #3 at C = 0.59 mg/mL; Figure S5: (a) CONTIN plot of sample PNVP-b-PVSt #3 at C = 0.257 mg/mL; (b) CONTIN plot of sample PNVP-b-PVSt #4 at C = 0.081 mg/mL; Figure S6: CONTIN plot of sample PNVP-b-PVSt #3 at C = 0.054 mg/mL; Figure S7: (a) UV-Vis spectra of PNVP-b-PVBu #1 with all concentrations of encapsulated curcumin; (b) UV-Vis spectra of PNVP-b-PVBu #2 with all concentrations of encapsulated curcumin; Figure S8; (a) UV-Vis spectra of PNVP-b-PVBu #3 with all concentrations of encapsulated curcumin; (b) UV-Vis spectra of PNVP-b-PVBu #4 with all concentrations of encapsulated curcumin; Figure S9; (a) UV-Vis spectra of PNVP-b-PVDc #1 with all concentrations of encapsulated cur-cumin; (b) UV-Vis spectra of PNVP-b-PVDc #2 with all concentrations of encapsulated curcumin; Figure S10: (a) UV-Vis spectra of PNVP-b-PVDc #3 with all concentrations of encapsulated cur-cumin; (b) UV-Vis spectra of PNVP-b-PVDc #4 with all concentrations of encapsulated curcumin; Figure S11: (a) UV-Vis spectra of PNVP-b-PVSt #1 with all concentrations of encapsulated curcumin; (b) UV-Vis spectra of PNVP-b-PVSt #2 with all concentrations of encapsulated curcumin; Figure S12: (a) UV-Vis spectra of PNVP-b-PVSt #3 with all concentrations of encapsulated curcumin; (b) UV-Vis spectra of PNVP-b-PVSt #4 with all concentrations of encapsulated curcumin; Figure S13: (a) UV-Vis spectra of indomethacin with various concentrations; (b) Calibration curve for indomethacin at 320 nm; Figure S14: (a) UV-Vis spectra of PNVP-b-PVBu #1 with all concentrations of encapsulated indomethacin; (b) UV-Vis spectra of PNVP-b-PVBu #3 with all concentrations of encapsulated indomethacin; Figure S15: UV-Vis spectra of PNVP-b-PVBu #4 with all concentrations of encapsulated indomethacin; Figure S16: (a) UV-Vis spectra of PNVP-b-PVDc #2 with all concentrations of encapsulated indomethacin; (b) UV-Vis spectra of PNVP-b-PVDc #4 with all concentrations of encapsulated indomethacin; Figure S17: (a) UV-Vis spectra of PNVP-b-PVSt #2 with all concentrations of encapsulated indomethacin; (b) UV-Vis spectra of PNVP-b-PVSt #4 with all concentrations of encapsulated indomethacin; Figure S18: UV-Vis spectra of PNVP-b-PVSt #5 with all concentrations of encapsulated indomethacin.
