# Supplementary material for: Amphiphilic Diblock Copolymers of Poly(N-vinyl pyrrolidone) and Poly(vinyl esters) Bearing N-Alkyl Side Chains for the Encapsulation of Curcumin and Indomethacin
Source: Polymers (Basel). 2025 Oct 26;17(21):2852. doi: 10.3390/polym17212852 (PMC12608385; doi:10.3390/polym17212852)
Supplement: Supplementary file 1 [file polymers-17-02852-s001.zip › polymers-3925008-supplementary.pdf]

# Amphiphilic Diblock Copolymers of Poly(N-vinyl pyrrolidone) and Poly(vinyl esters) Bearing N-Alkyl Side Chains for the Encapsulation of Curcumin and Indomethacin

**Nikolaos V. Plachouras, Aikaterini-Maria Gkolemi, Alexandros Argyropoulos, Athanasios Bouzoukas, Theodosia-Panagiota Papazoglou, Nikoletta Roka and Marinos Pitsikalis ***

Industrial Chemistry Laboratory, Department of Chemistry, National and Kapodistrian University of Athens, Panepistimiopolis Zografou, 15771 Athens, Greece; plachouras.v.nikolaos@gmail.com (N.V.P.); katerinagolemi228@gmail.com (A.-M.G.); argiropoulosal@gmail.com (A.A.); thanasisbuzukas@gmail.com (A.B.); pnpapazoglou@gmail.com (T.-P.P.); nikolettaroka@yahoo.gr (N.R.)

* Correspondence: pitsikalis@chem.uoa.gr; Tel.: +30-210-727-4768

# Supporting Information Section

**Supplementary Material File S1. Synthesis of the PNVP-b-PVEs block copolymers**

The synthesis of the PNVP-b-PVEs block copolymers was accomplished via RAFT polymerization and sequential addition of monomers. Since both types of monomers have low reactivities the same CTA can be employed for the synthesis of well-defined block copolymers. In the present study O-ethyl S-(phthalimidylmethyl) xanthate was used as the CTA, since it is well-known that it promotes very good control over the RAFT polymerization of Less Activated Monomers. In particular, it has been efficiently employed for the homopolymerization of both NVP and VEs in the past. The procedure for the synthesis of the PNVP-b-PVEs block copolymers is depicted in the following Scheme S1.

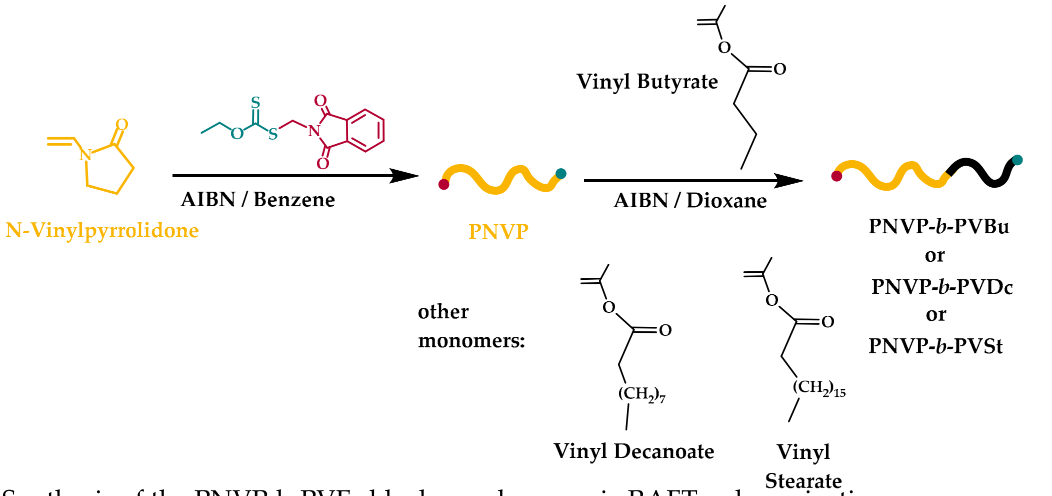


**Scheme S1**. Synthesis of the PNVP-b-PVEs block copolymers via RAFT polymerization.

NVP was polymerized first providing well-defined macromolecular CTAs (macro-CTAs) having controlled molecular characteristics, ie. relatively narrow molecular weight distribution and molecular weight, which was close to the stoichiometric values. In order to better control the dispersity values and to achieve quantitative incorporation of the CTA's moieties as the polymer's end-groups the polymerization was not allowed to proceed to very high yields. In most cases the reaction was terminated by cooling the reaction flask when the conversion was less than 80% in all cases. At higher yields the solution becomes very viscous and the system becomes more or less heterogeneous leading to increased dispersity values. In addition, upon increasing the polymerization yield the possibility to lose the control over the end-groups is increased. The PNVP macro-CTAs were precipitated, re-dissolved and re-precipitated to remove unreacted monomer. The samples were finally dried in a vacuum oven.

The purified and dried macro-CTAs were further employed for the polymerization of various VEs, namely VBu, VDc and VSt. In order to minimize the viscosity problems, the termination and other side reactions involved in RAFT radical polymerization, thus achieving the best degree of control during the copolymerization reaction, the conversion of the polymerization of VEs was not allowed to reach high values, i.e. less than 50% in most cases. The efficiency of this approach was monitored by SEC and $^1$H NMR measurements. Characteristic SEC traces from the synthesis of the block copolymers and a typical $^1$H NMR spectrum are provided in the following Figures.

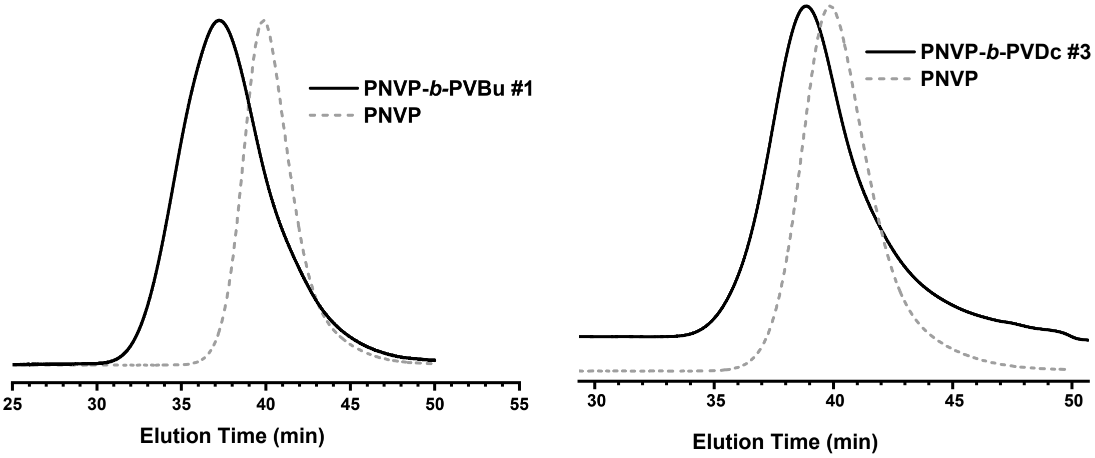


**Figure.** SEC traces from the synthesis of the block copolymers PNVP-*b*-PVBu #1 and PNVP-*b*-PVDc #3.

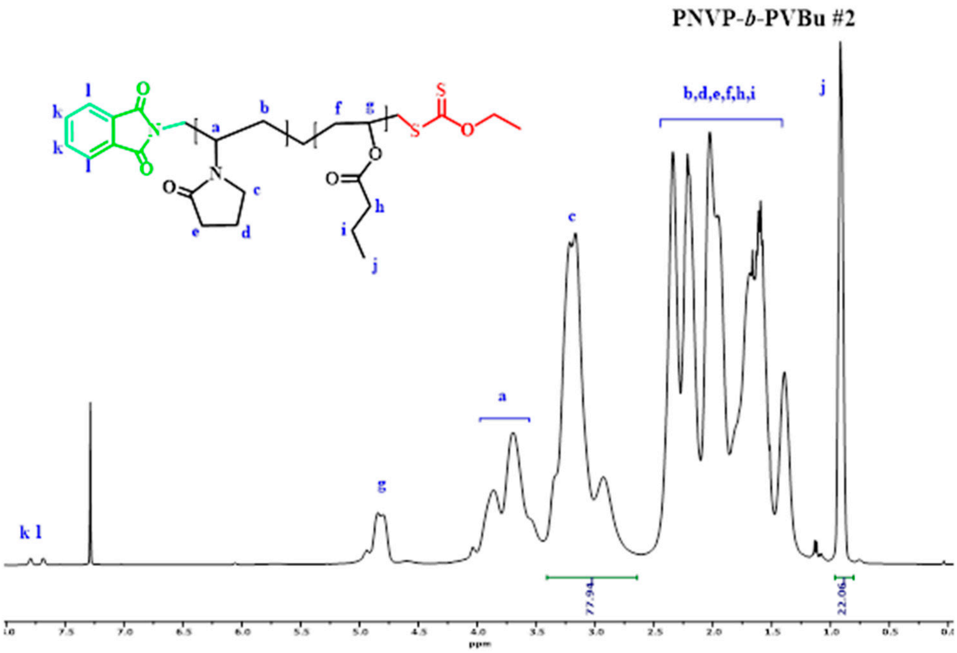


**Figure**. ¹H NMR spectrum of the block copolymer PNVP-b-PVBu #2.

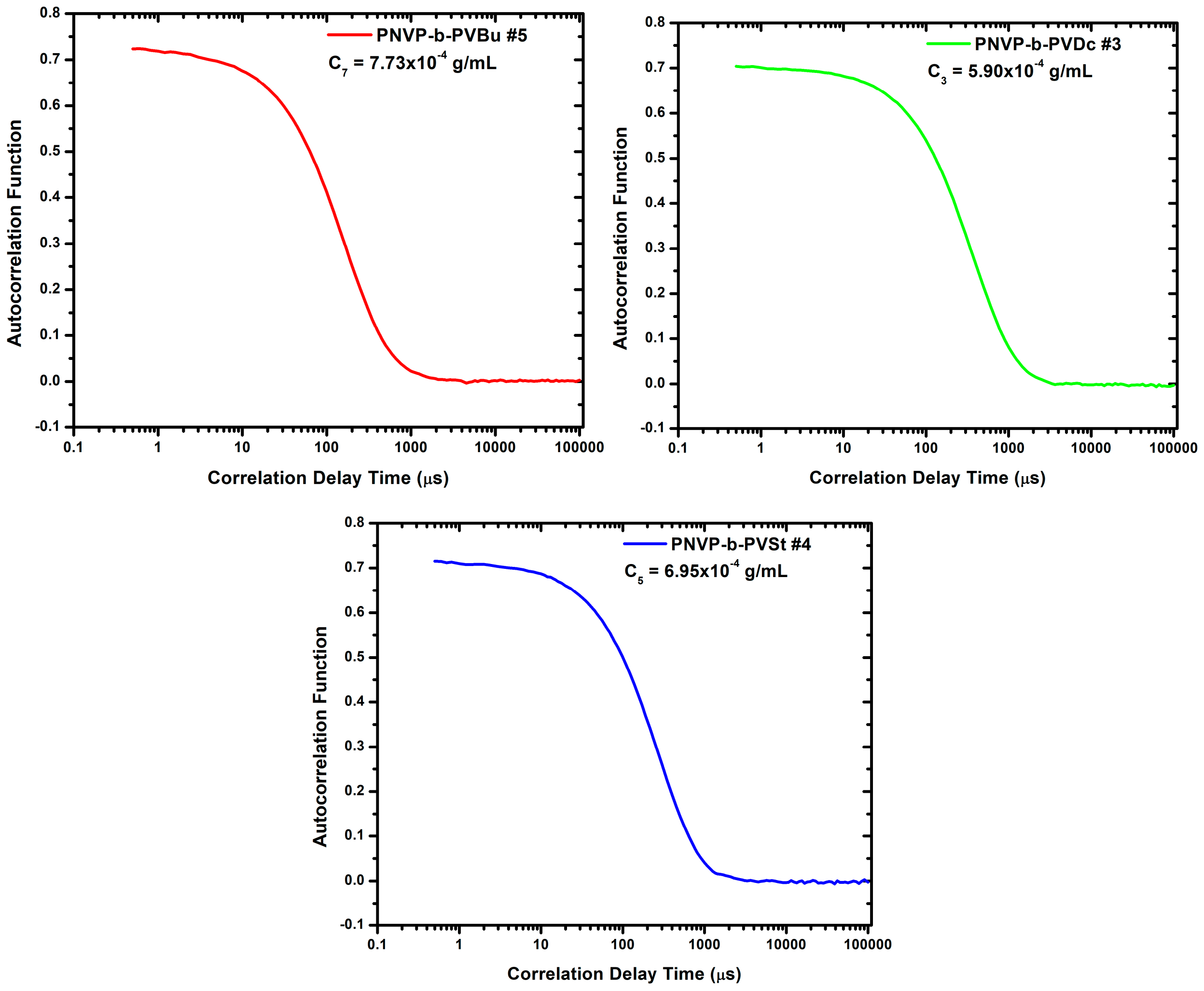


**Figure S1**. Autocorrelation functions from DLS experiments from various block copolymer micellar aqueous solutions.

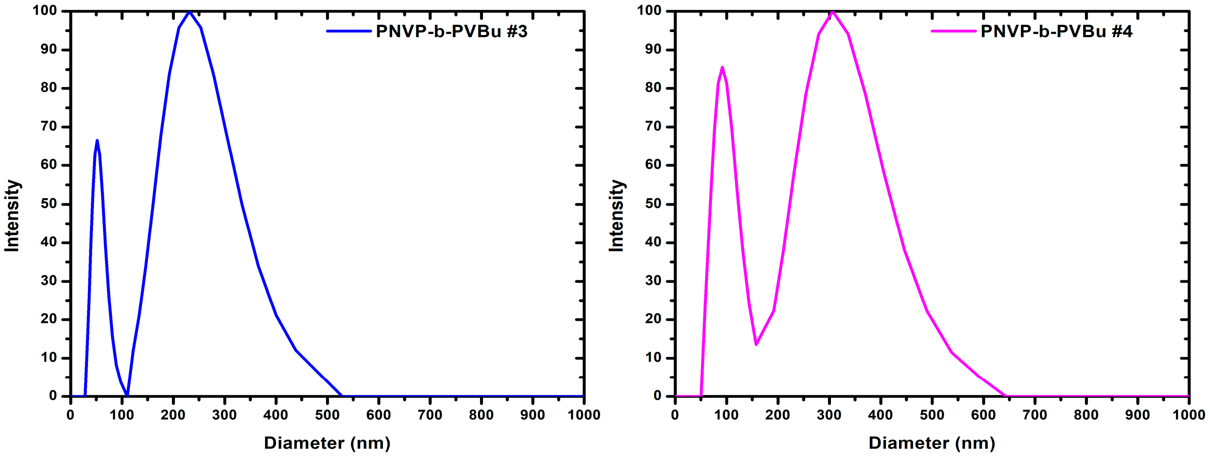


(a) (b)

**Figure S2.** (a) CONTIN plot of sample PNVP-b-PVBu #3 at C= 0.093 mg/mL; (b) CONTIN plot of sample PNVP-b-PVBu #4 at C= 0.758 mg/mL.

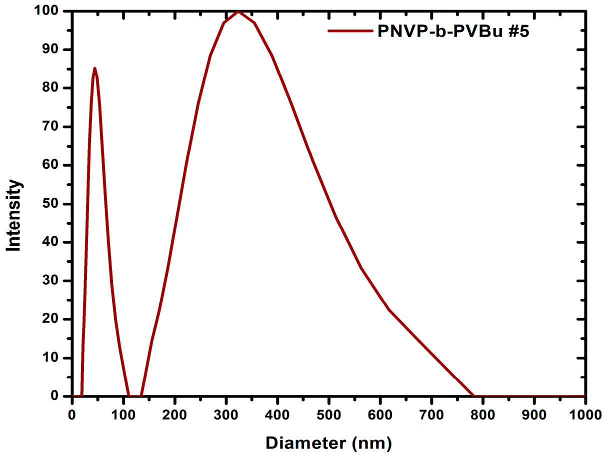


**Figure S3.** CONTIN plot of sample PNVP-b-PVBu #5 at C= 0.095 mg/mL.

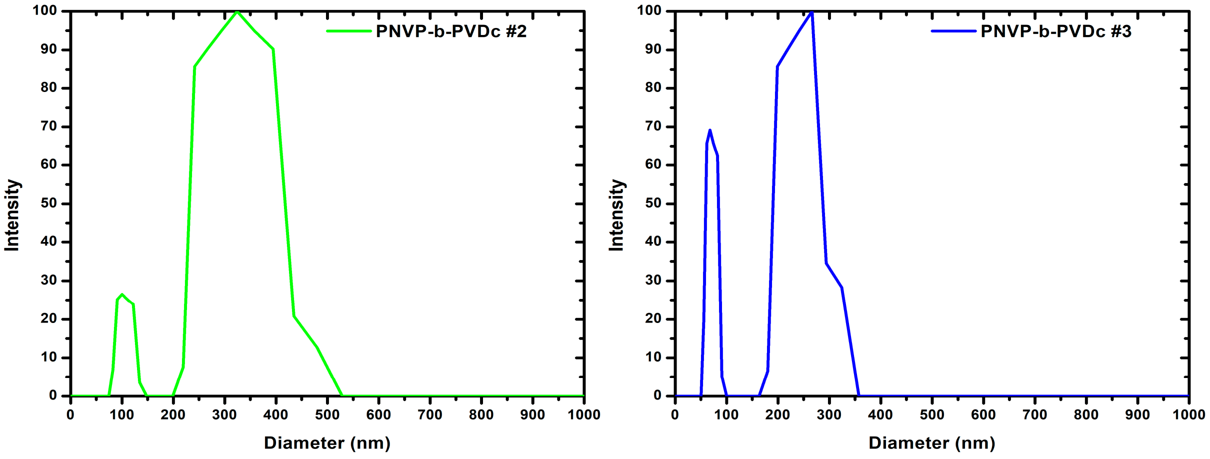


(a) (b)

**Figure S4.** (a) CONTIN plot of sample PNVP-b-PVDc #2 at C= 0.659 mg/mL; (b) CONTIN plot of sample PNVP-b-PVDc #3 at C= 0.59 mg/mL.

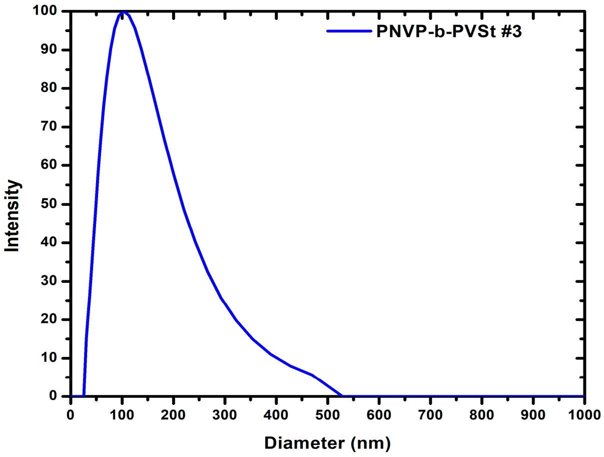


(**a**)

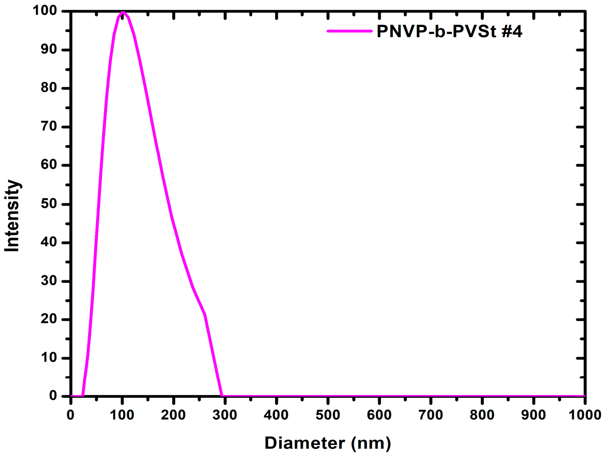


(**b**)

**Figure S5.** (a) CONTIN plot of sample PNVP-b-PVSt #3 at C= 0.257 mg/mL; (b) CONTIN plot of sample PNVP-b-PVSt #4 at C= 0.081 mg/mL.

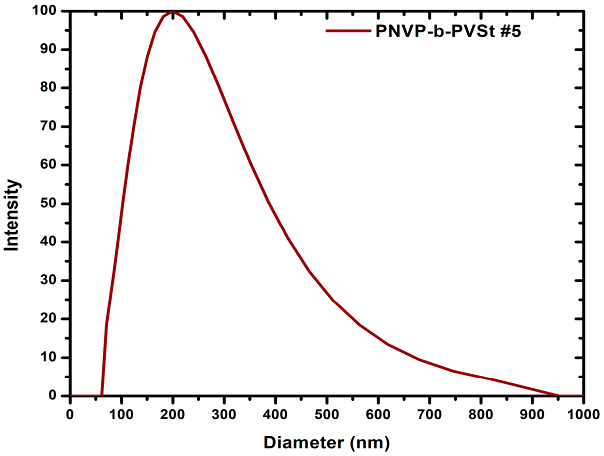


**Figure S6.** CONTIN plot of sample PNVP-b-PVSt #3 at C= 0.054 mg/mL.

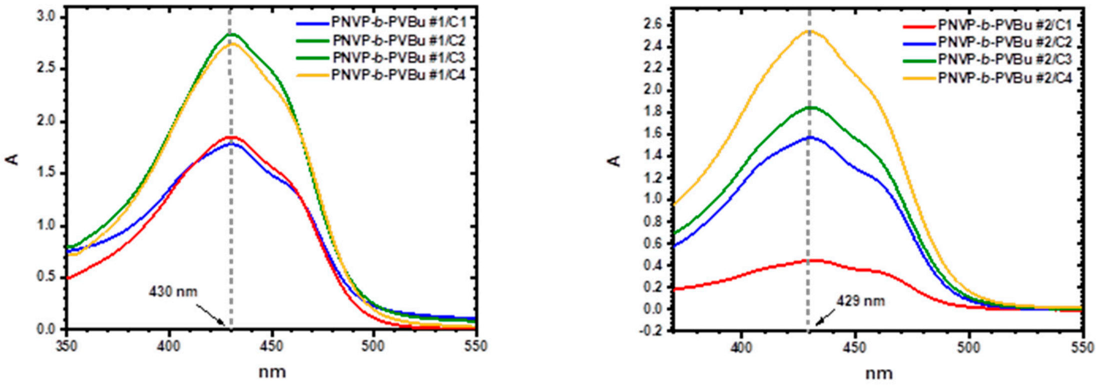


(**a**) (**b**)

**Figure S7.** (a) UV-Vis spectra of PNVP-b-PVBu #1 with all concentrations of encapsulated curcumin; (b) UV-Vis spectra of PNVP-b-PVBu #2 with all concentrations of encapsulated curcumin.

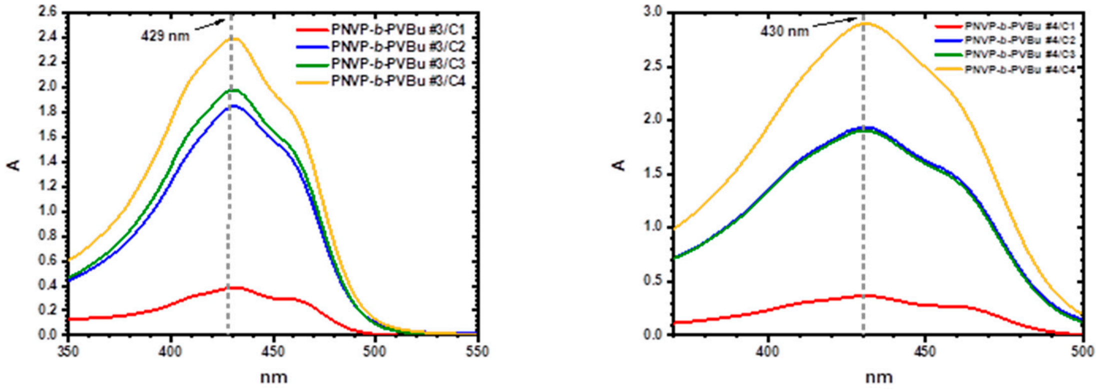


(**a**) (**b**)

**Figure S8.** (a) UV-Vis spectra of PNVP-b-PVBu #3 with all concentrations of encapsulated curcumin; (b) UV-Vis spectra of PNVP-b-PVBu #4 with all concentrations of encapsulated curcumin.

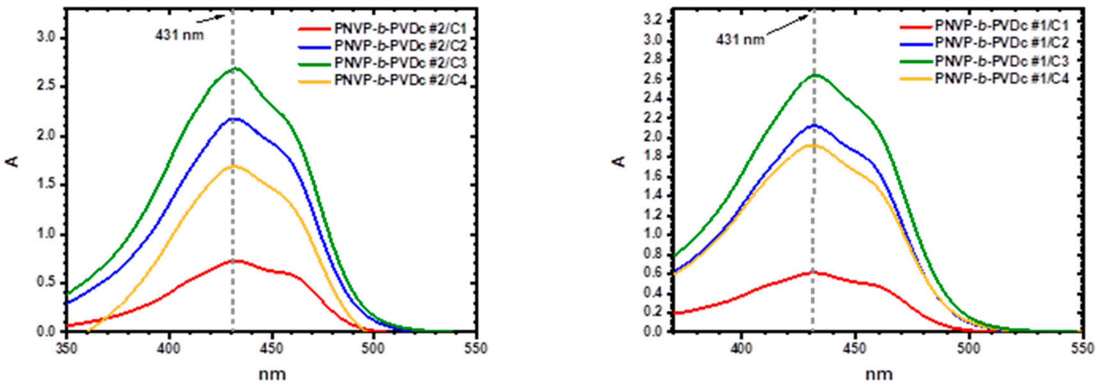


(**a**) (**b**)

**Figure S9.** (a) UV-Vis spectra of PNVP-b-PVDc #1 with all concentrations of encapsulated curcumin; (b) UV-Vis spectra of PNVP-b-PVDc #2 with all concentrations of encapsulated curcumin.

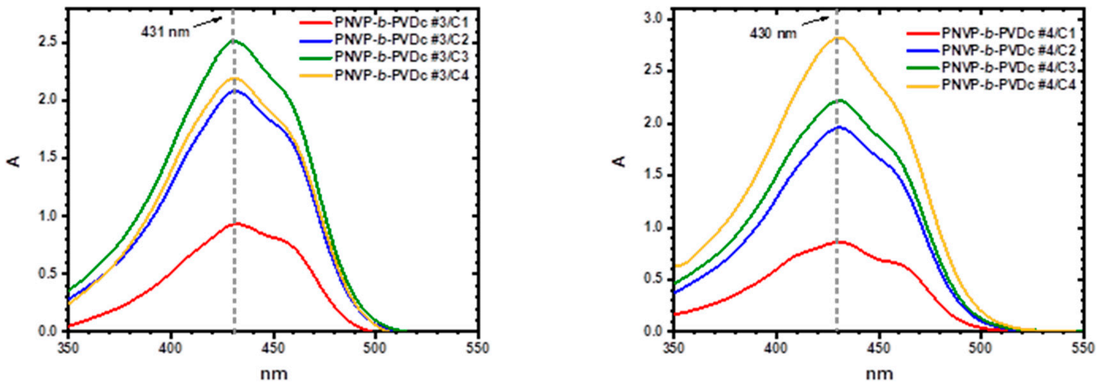


(**a**) (**b**)

**Figure S10.** (a) UV-Vis spectra of PNVP-b-PVDc #3 with all concentrations of encapsulated curcumin; (b) UV-Vis spectra of PNVP-b-PVDc #4 with all concentrations of encapsulated curcumin.

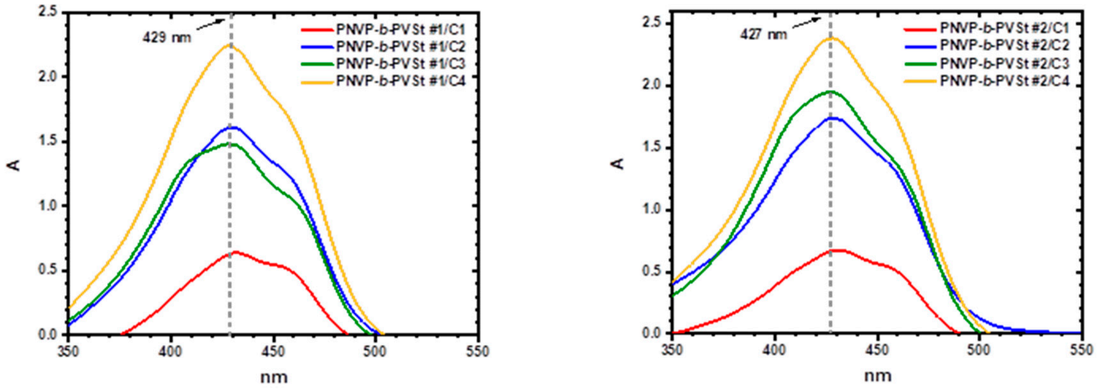


(**a**) (**b**)

**Figure S11.** (a) UV-Vis spectra of PNVP-b-PVSt #1 with all concentrations of encapsulated curcumin; (b) UV-Vis spectra of PNVP-b-PVSt #2 with all concentrations of encapsulated curcumin.

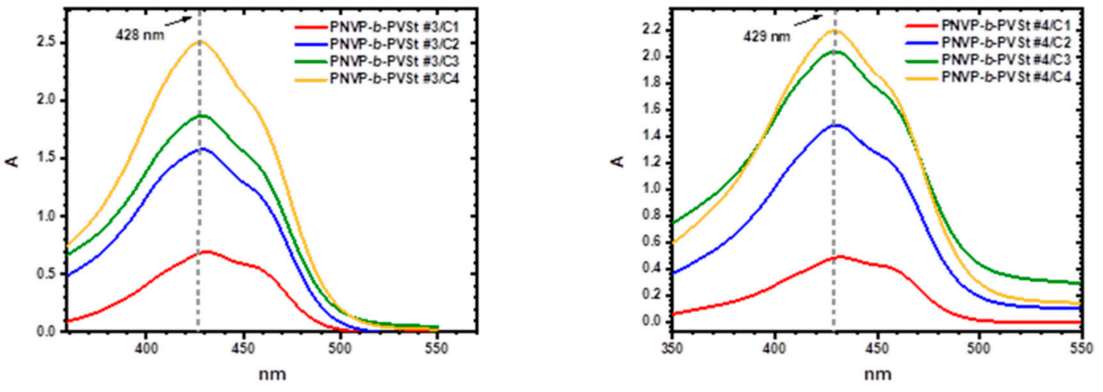


(**a**) (**b**)

**Figure S12.** (a) UV-Vis spectra of PNVP-b-PVSt #3 with all concentrations of encapsulated curcumin; (b) UV-Vis spectra of PNVP-b-PVSt #4 with all concentrations of encapsulated curcumin.

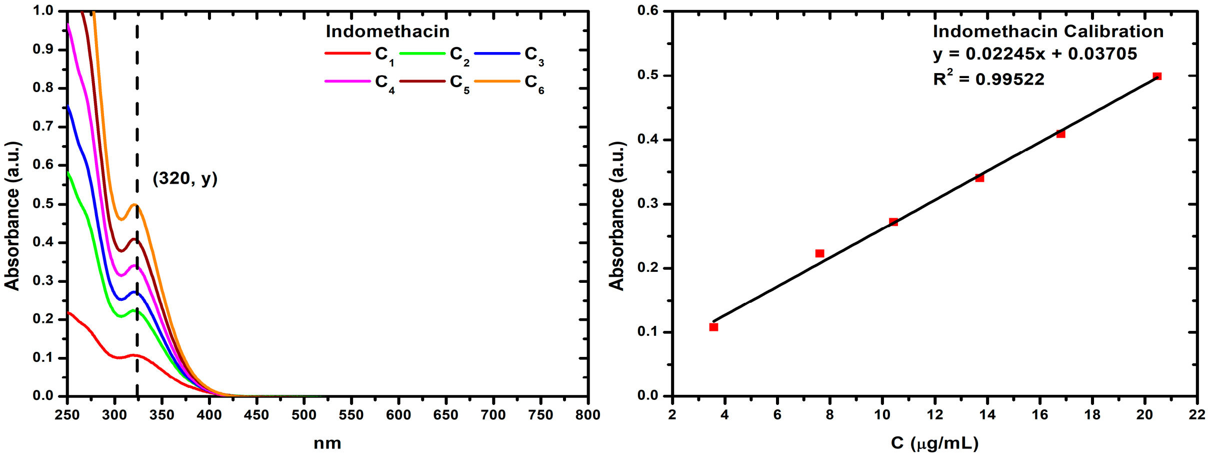


(**a**) (**b**)

**Figure S13.** (a) UV-Vis spectra of indomethacin with various concentrations; (b) Calibration curve for indomethacin at 320 nm.

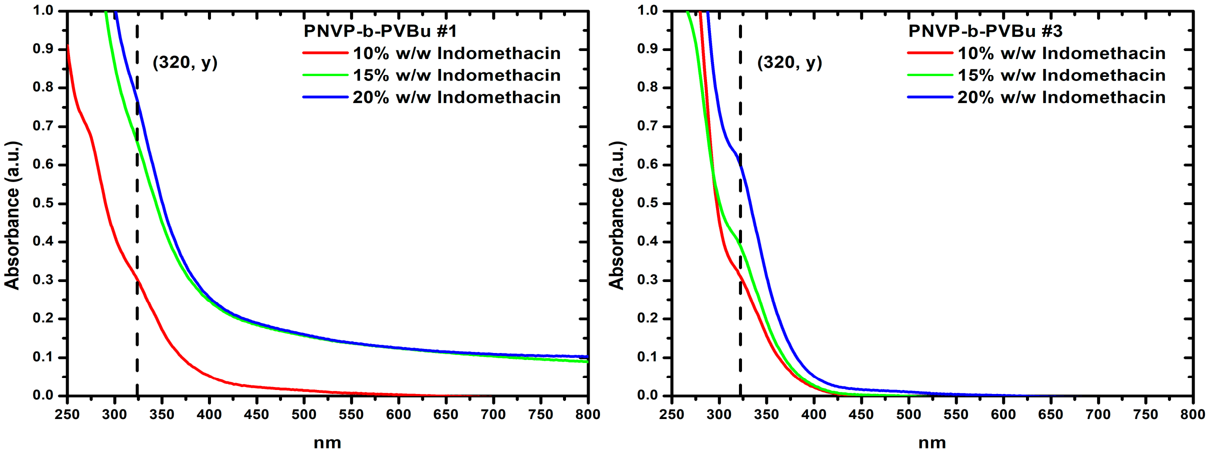


(**a**) (**b**)

**Figure S14.** (a) UV-Vis spectra of PNVP-b-PVBu #1 with all concentrations of encapsulated indomethacin; (b) UV-Vis spectra of PNVP-b-PVBu #3 with all concentrations of encapsulated indomethacin.

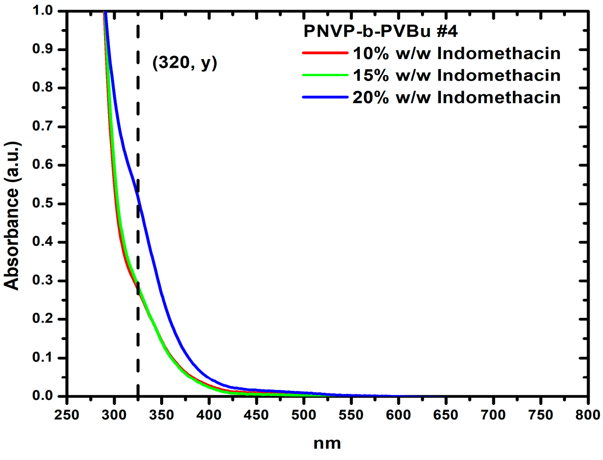


**Figure S15.** UV-Vis spectra of PNVP-b-PVBu #4 with all concentrations of encapsulated indomethacin.

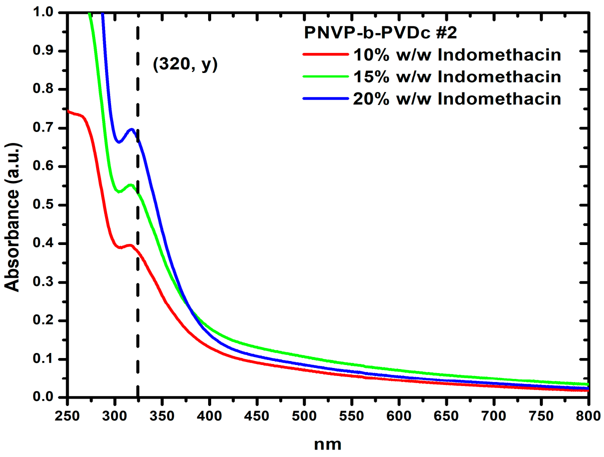


(**a**)

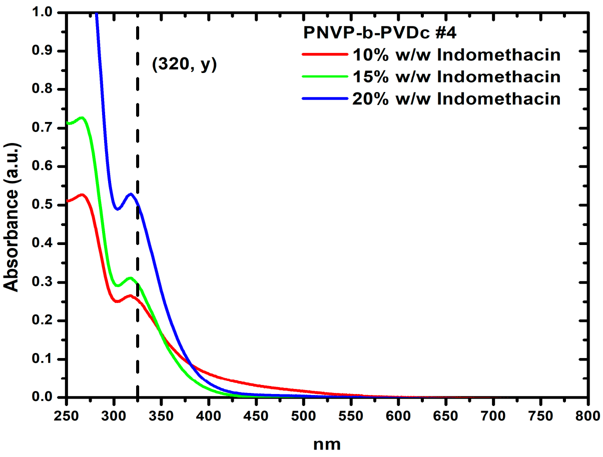


(**b**)

**Figure S16.** (a) UV-Vis spectra of PNVP-b-PVDc #2 with all concentrations of encapsulated indomethacin; (b) UV-Vis spectra of PNVP-b-PVDc #4 with all concentrations of encapsulated indomethacin.

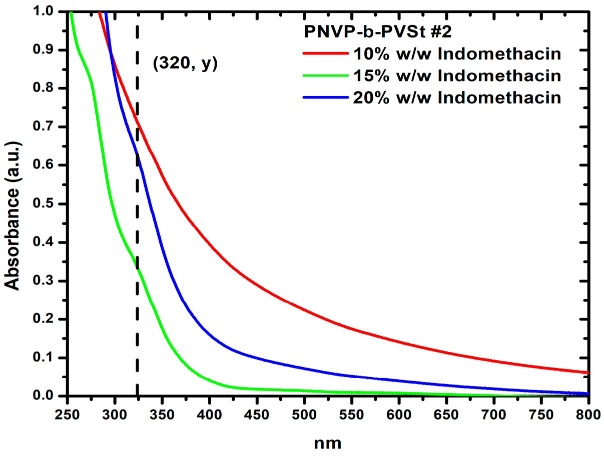


(**a**)

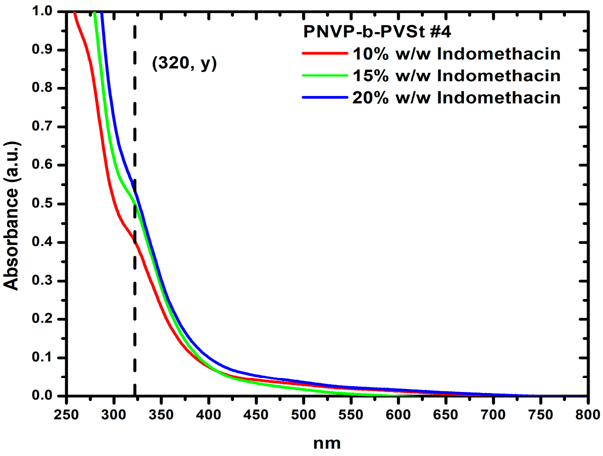


(**b**)

**Figure S17.** (a) UV-Vis spectra of PNVP-b-PVSt #2 with all concentrations of encapsulated indomethacin; (b) UV-Vis spectra of PNVP-b-PVSt #4 with all concentrations of encapsulated indomethacin.

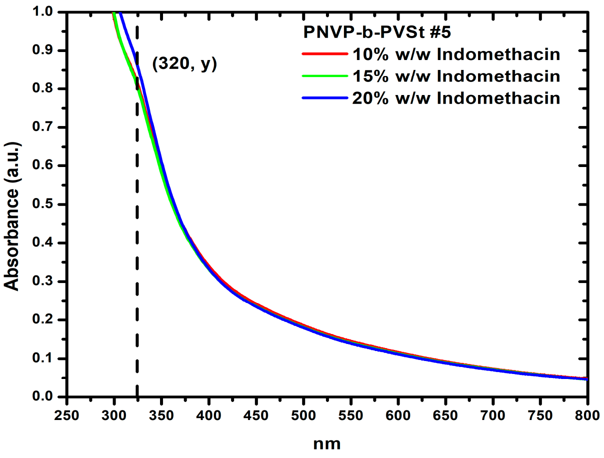


**Figure S18.** UV-Vis spectra of PNVP-b-PVSt #5 with all concentrations of encapsulated indomethacin.
